# Structural brain alterations in chronic primary pain: a multimodal MRI study

**DOI:** 10.1016/j.nicl.2026.104023

**Published:** 2026-06-19

**Authors:** Salome Häuselmann, Anna Wyss, Nina Bischoff, Rupert Bruckmaier, Josef Gross, Chantal Berna, Martin grosse Holtforth, Selma Aybek, Nicolas Gninenko

**Affiliations:** a Psychosomatic Medicine, Department of Neurology, Inselspital, Bern University Hospital, University of Bern, Switzerland; b Graduate School of Cellular and Biomedical Sciences (GCB), University of Bern, Switzerland; c Translational Imaging Center (TIC), Swiss Institute for Translational and Entrepreneurial Medicine, Bern, Switzerland; d Veterinary Physiology, Vetsuisse Faculty, University of Bern, Switzerland; e Center for Integrative and Complementary Medicine, Department of Anesthesiology, Lausanne University Hospital, Switzerland; f Department of Psychology, University of Bern, Switzerland; g Department of Neurology, Faculty of Science and Medicine, University of Fribourg, Switzerland

**Keywords:** Chronic primary pain, Cortical thickness, Fractal dimension, Gyrification, Structural connectivity, Sulcal depth, Surface-based morphometry

## Abstract

Chronic primary pain (CPP) occurs without an identifiable causal disease and is characterized by persistent pain, emotional distress and functional impairment. Persistent pain may be accompanied by structural brain alterations linked to chronification. We investigated cortical surface morphometry and structural connectivity in CPP and explored associations with related biopsychosocial characteristics.

Thirty patients with CPP and 30 matched healthy controls (HCs) underwent psychometric assessment, pain sensitivity testing, salivary sampling (cortisol, α-amylase), and MRI (T1-weighted, diffusion-weighted imaging (DWI)). Surface-based morphometry features (SBM) were estimated from T1-weighted data across multiple brain parcellations. DWI was used to reconstruct weighted structural connectomes via probabilistic tractography and to compute node-level graph-theoretical metrics. Partial least squares correlation (PLSC) assessed multivariate associations between imaging metrics and biopsychosocial characteristics.

Compared with HCs, CPP patients showed focal cortical alterations dominated by folding-related features consistent across different brain parcellation schemes: increased gyrification in left prefrontal regions; reduced sulcal depth in right lateral frontal and orbitofrontal and right medial temporo-occipital regions; and reduced fractal dimension in posterior cingulate regions. Structural connectivity metrics showed only trend-level group differences that did not survive correction for multiple comparisons.

PLSC revealed significant covariation between distributed SBM patterns and biopsychosocial characteristics, including perceived stress, childhood trauma, and α-amylase concentration, mainly expressed in HCs, linking stress-related features to profiles of gyrification, sulcal depth, and fractal dimension. These findings suggest that cortical surface morphology may be structurally altered in patients with CPP and encourage further longitudinal studies.

## Highlights

• Increased gyrification in left prefrontal regions in chronic primary pain (CPP)
• CPP patients show reduced sulcal depth in right frontal and temporal regions
• Reduced folding complexity in posterior cingulate regions in CPP
• No significant node-level white matter connectivity differences (CPP vs. controls)
• In controls, stress and childhood adversity covaried with the cortical patterns

## 1 Introduction

Chronic pain is a complex and debilitating condition that affects approximately 21% of the European population (Rometsch et al., 2025). Biological, psychological, and social factors jointly contribute to the development and maintenance of chronic pain, yet the exact neural mechanisms remain elusive (Gatchel et al., 2007; Bevers et al., 2016; Kaplan et al., 2024; Fillingim et al., 2025). Chronic primary pain (CPP) refers to persistent pain that cannot be better accounted for by an identifiable causal disease or tissue pathology and is accompanied by emotional distress and functional disability (World Health Organization, 2022). CPP encompasses conditions such as chronic widespread pain (including fibromyalgia), complex regional pain syndrome, and chronic primary headache or orofacial pain (Nicholas et al., 2019). Converging evidence suggests that alterations in brain structure and function may contribute to the persistence of pain even in the absence of ongoing peripheral damage (Kaplan et al., 2024; Quidé et al., 2024). Accordingly, advances in neuroimaging have provided important tools to investigate the neurobiological underpinnings of CPP and to characterize neural correlates of this inherently subjective experience (Kaplan et al., 2024; Bautin et al., 2025; van der Miesen et al., 2019).

Previous research in CPP has mostly focused on gray matter morphology (see review by Xin et al., 2023) and functional connectivity (see review by Cavicchioli et al., 2025), whereas studies of cortical surface-based morphometry and white matter structural connectivity remain limited and methodologically heterogeneous (Bautin et al., 2025; van der Miesen et al., 2019). Addressing this gap is relevant because white matter architecture provides the anatomical pathways for long-range communication between brain regions and supports integration across large-scale functional networks (Forkel et al., 2022). In addition, cortical surface features derived from surface-based morphometry (e.g., cortical thickness, gyrification) capture complementary aspects of cortical architecture (Dahnke et al., 2013) that may be relevant for CPP and its affective dimensions, given evidence that cortical geometry relates to brain function and behavior (Schwartz et al., 2023). Finally, emerging work suggests that white matter tract organization and cortical geometry are coupled, pointing to a dynamic interplay between structural connectivity and cortical surface architecture (Li et al., 2025a).

Studies of cortical surface features in CPP have most often focused on cortical thickness, with findings across CPP conditions frequently, though inconsistently, suggesting reductions in brain areas commonly involved in nociceptive and affective-cognitive pain processing (Henn et al., 2023; Chaudhry et al., 2025; Neto et al., 2024; Jensen et al., 2013; Lee et al., 2015; Magon et al., 2018; Li et al., 2025b; Liu et al., 2024). A meta-analysis indicates reduced cortical thickness in the left precentral gyrus in chronic pain relative to healthy controls (HCs), though this was only observed in a secondary exploratory analysis using threshold-free cluster enhancement (Henn et al., 2023). Another meta-analysis comparing patients with chronic pain, major depressive disorder, and anxiety disorders with HCs found convergent reductions in cortical thickness across these disorders in the right insula, left anterior cingulate cortex, triangular part of the left inferior frontal gyrus, and left middle temporal gyrus (Yu et al., 2025). However, much of the literature remains fragmented, with many studies focusing on a single CPP condition (e.g., fibromyalgia, complex regional pain syndrome, migraine, temporomandibular joint disorders), leaving it unclear whether observed neuroanatomical alterations reflect condition-specific signatures or generalize across CPP conditions (Neto et al., 2024; Jensen et al., 2013; Lee et al., 2015; Magon et al., 2018; Li et al., 2025b; Liu et al., 2024). Only a few studies have examined surface metrics beyond cortical thickness; for example, a chronic shoulder pain study assessed cortical thickness, gyrification, and sulcal depth and reported no group differences in cortical thickness or gyrification, whereas patients compared to HCs showed shallower sulcal depth across regions including the right central sulcus, posterior insula, inferior frontal and dorsomedial prefrontal cortices, precuneus, middle temporal cortex, as well as the left medial orbitofrontal cortex (Niddam et al., 2019). Additionally, regional sulcal depths of the right central sulcus and the left medial orbitofrontal cortex were correlated with pain-related measures, such as pain intensity, severity, and duration (Niddam et al., 2019).

While cortical surface features characterize the morphology of cortical regions, diffusion-weighted imaging (DWI) provides complementary information about the white matter pathways that support structural connectivity between regions by quantifying water diffusion in tissue (Stejskal and Tanner, 1965). A range of DWI-derived indices and modeling approaches have been used to characterize white matter structural connectivity in CPP, and a recent review by Bautin et al. (2025) highlights both methodological heterogeneity and inconsistent findings across CPP cohorts. Beyond local microstructural metrics such as fractional anisotropy (FA), DWI also enables construction of tractography-based brain networks for structural connectomics and subsequent graph-theoretical analysis, in which gray matter regions are represented as nodes connected by white matter pathways (edges) (Bautin et al., 2025). Studies in single-condition cohorts suggest alterations in structural network topology in CPP, reflected in altered network connectivity, integration, and segregation features (Bautin et al., 2025; Silvestro et al., 2021; Kurokawa et al., 2021; Wada et al., 2017; Huang et al., 2020; Pijnenburg et al., 2016; Mei et al., 2024; Liu et al., 2017; Matoso et al., 2024; Rubinov and Sporns, 2010).

Cortical surface architecture and white matter network organization have rarely been examined jointly in CPP. Therefore, it remains unclear whether multimodal structural alterations converge on shared patterns across CPP conditions and how such patterns relate to clinical characteristics.

To address this gap, we combined surface-based morphometry with DWI-based connectomics in a sample of 30 patients with CPP and 30 HCs. Our primary research questions were to identify whether patients with CPP differ from HCs in (1) cortical surface-based morphometric features, including cortical thickness, gyrification, sulcal depth, and fractal dimension, and in (2) white matter structural connectivity, assessed using probabilistic tractography and graph-theoretical metrics derived from FA structural connectomes. Based on prior evidence of structural brain alterations in chronic pain, we hypothesized that CPP patients would show altered cortical surface features and structural connectivity relative to HCs, particularly in regions implicated in sensory-discriminative and affective-cognitive pain processing. As a secondary exploratory objective, we aimed to examine whether multimodal structural alterations were associated with CPP-related biopsychosocial characteristics, including stress biomarkers, perceived stress, childhood adversity, pain sensitivity, symptom duration, and pain burden.

## 2 Materials and methods

### 2.1 Participants

The study was performed at the University Hospital Inselspital in Bern, Switzerland. Thirty patients with the principal diagnosis of chronic primary pain (CPP; ICD-11: MG30.0) and thirty healthy controls (HCs), matched for age and sex, were recruited between 2023 and 2024. Exclusion criteria included major neurological or severe psychiatric disorders, inability to adhere to study procedures, or contraindications for MRI (e.g., implanted medical devices, metal implants, pregnancy). Assessments were scheduled relative to MRI acquisition as follows: demographic and clinical variables and stress-related self-report questionnaires were collected approximately one week before MRI; mood and pain-related questionnaires were collected on the day of MRI; salivary cortisol was collected on the preceding day, salivary α-amylase immediately before and after MRI, and peg algometry after MRI. Detailed descriptions of these procedures are provided in Supplementary Material A.1 - A.3 and in (Häuselmann et al., 2026). The study was approved by the Ethics Committee of the Canton of Bern (SNCTP000004529, 2020–02283) and conducted in accordance with the principles of the Declaration of Helsinki. The protocol was preregistered on ClinicalTrials.gov (NCT05086380). All participants provided written informed consent and were reimbursed for travel expenses.

### 2.2 MRI data acquisition

MRI data were acquired on a 3 Tesla scanner (Magnetom Prisma, Siemens Healthineers, Germany). During image acquisition (anatomical and DWI), participants either watched an animal documentary or kept their eyes closed.

#### 2.2.1 Anatomical imaging

T1-weighted structural imaging was performed using a sagittally oriented 3D magnetization-prepared rapid acquisition gradient echo (MPRAGE) sequence (GRAPPA, acceleration factor = 2; phase encoding direction = anterior-to-posterior) with the following parameters: repetition time (TR) = 2330 ms, echo time (TE) = 3.03 ms, inversion time (TI) = 1100 ms, flip angle (FA) = 8°, matrix size = 256 × 256 × 176, and isotropic voxel size = 1 mm^3^.

##### 2.2.1.1 Surface-based morphometry (SBM)

**Fig. 1:**
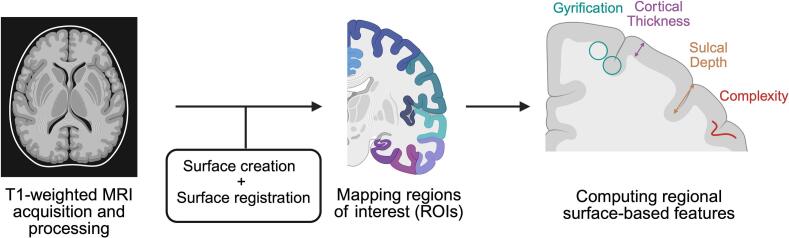
Surface-based morphometry workflow for ROI-wise cortical surface feature extraction. T1-weighted images were processed using the standard CAT12 surface-based morphometry pipeline. Cortical surface reconstruction was performed with the projection-based thickness method, which jointly estimates cortical thickness and the central surface. Subject-specific central surfaces were registered to the corresponding hemisphere of the FreeSurfer *fsaverage* template. Surface atlases were mapped to individual surfaces using the spherical registration parameters obtained during surface processing. Surface-based measures (e.g., cortical thickness, sulcal depth) were then extracted for each ROI in native space. Created in BioRender. Häuselmann, S. (2026) https://BioRender.com/4zyhoc8.

T1-weighted images were processed using the standard surface-based morphometry pipeline from the Computational Anatomical Toolbox (CAT12.8.1 r2043) and SPM12 (Wellcome Centre for Human Neuroimaging, University College London, UK) within MATLAB (R2023a; The MathWorks Inc., Natick, MA, USA) for surface-based morphometry (SBM) analysis (Fig. 1). Preprocessing included denoising, resampling, bias-field correction, and affine registration, followed by tissue segmentation into gray matter, white matter, and cerebrospinal fluid. Skull stripping was performed using the adaptive probability region-growing method. Tissue segments were spatially normalized to the CAT12 MNI template space via Geodesic Shooting registration. Cortical thickness and central surfaces were estimated simultaneously using a projection-based thickness method, followed by topological correction and surface refinement, before registration to the FreeSurfer *fsaverage* template (Dahnke et al., 2013).

From these surfaces, maps of cortical thickness (Dahnke et al., 2013), gyrification index (Dahnke et al., 2013; Luders et al., 2006), sulcal depth (Dahnke et al., 2013), and cortical complexity (i.e., fractal dimension) (Yotter et al., 2011) were computed for each subject on a point-wise (i.e., vertex-wise) or ROI-wise level (Gaser et al., 2024). All surface maps were resampled to the standard CAT12 surface template and smoothed with a 15 mm full-width at half-maximum (FWHM) Gaussian kernel for cortical thickness and 20 mm FWHM for gyrification, cortical complexity, and sulcal depth to increase signal-to-noise ratio and account for inter-individual variability. Additionally, sulcal depth values were square root transformed before statistical analysis to improve normality. ROI-specific mean surface values were extracted in native space using the Desikan–Killiany (DK40) (Desikan et al., 2006), Destrieux (Destrieux et al., 2010), and Schaefer atlases (Schaefer et al., 2018) prior to spatial normalization (Gaser et al., 2024).

Cortical thickness was defined as the distance between the white matter surface and the pial surface, capturing the width of the cortical gray matter ribbon. Gyrification quantified local cortical folding and was computed from the absolute mean curvature of the central cortical surface, with higher values indicating a more strongly folded cortex. Sulcal depth was defined as the distance from the central surface to its enclosing hull, such that vertices located deeper within sulci had larger depth values. Cortical complexity was quantified as the local fractal dimension of the central surface, estimated from spherical-harmonic reconstructions across multiple spatial scales (Gaser et al., 2024).

##### 2.2.1.2 Statistical analysis for SBM

To test whether patients with CPP differ from HCs in cortical surface morphology, we performed both vertex-wise and ROI-wise group comparisons of cortical thickness, gyrification, sulcal depth, and cortical complexity. Vertex-wise statistical analyses were performed on the smoothed surface maps, and significant clusters were anatomically labeled based on their overlap with regions of the DK40 and Destrieux atlases. In a complementary ROI analysis, mean surface values for each measure were extracted from all DK40, Destrieux, and Schaefer atlas regions for subsequent statistical testing.

For vertex-wise analyses, a general linear model (GLM) with group as the between-subject factor and age, sex, and mood composite score (average of z-scored STAI-2 and BDI, due to their high intercorrelation (Spearman's ρ > 0.88)) as covariates of no interest was implemented as a two-sample *t*-test in SPM12 to compare between groups. Multiple comparisons were controlled using cluster-level family-wise error (FWE) correction across the cortical surface, and a cluster-level FWE-corrected *p* < 0.05 was considered statistically significant. For the ROI-wise analyses, mean ROI values were entered into equivalent GLMs; *p-*values were adjusted for multiple comparisons using the Holm-Bonferroni correction, and Holm-Bonferroni-corrected *p* < 0.05 was considered statistically significant. Additionally, a sensitivity analyses with reduced (excluding the mood composite score) and extended (additionally correcting for psychotropic medication) covariate adjustments of the ROI-wise analyses are presented in Supplementary Material B.4. Vertex-wise and ROI-wise results were visualized on the *fsaverage* surface.

#### 2.2.2 Diffusion-weighted imaging

DWI was performed using an interleaved echo-planar spin-echo (EPSE) sequence (acceleration factor = 2, phase encoding direction = anterior-to-posterior) with the following parameters: TR = 3700 ms, TE = 87 ms, matrix size = 96 × 96 × 56 (no gap), isotropic voxel size = 2.2 mm^3^, echo spacing = 0.58 ms and bandwidth = 2004 Hz/Px. Diffusion weighting was applied along 30 directions (b_max=30_ = 3000 s/mm^2^), and three non-diffusion-weighted (b_0_ = 0 s/mm^2^) images were acquired and averaged in k-space. Processing steps were performed using SPM12 (Wellcome Centre for Human Neuroimaging, University College London, UK) implemented in MATLAB R2023a (The MathWorks Inc., Natick, MA, USA), FreeSurfer v7.4.1 (surfer.nmr.mgh.harvard.edu), FSL v6.0.5.2 (FMRIB Software Library; fsl.fmrib.ox.ac.uk/fsl) (Jenkinson et al., 2012) and MRtrix3 v3.0.3 (mrtrix.org) (Tournier et al., 2019). Detailed descriptions of preprocessing, structural connectome reconstruction, and node-level graph-theoretical analyses are provided in the Supplementary Material A.4. To test whether CPP is associated with altered white matter structural connectivity, we examined group differences in node-level graph-theoretical metrics derived from FA structural connectomes. Node-level features of structural white matter network organization were adjusted for age and sex in a primary model, and subsequently also for the mood composite score in a secondary model.

### 2.3 Associations between imaging and clinical measures

To address whether multimodal structural alterations were associated with CPP-related biopsychosocial characteristics, we conducted a PLSC analysis using the publicly available MATLAB-based PLS toolbox (https://github.com/FND-ResearchGroup/myPLS_SL) (Zöller et al., 2019). This method identifies latent variables that optimally capture the covariance between two multivariate datasets by computing weighted linear combinations of morphometric variables and CPP-related biopsychosocial characteristics (Krishnan et al., 2011; McIntosh and Lobaugh, 2004). The weights (saliences) reflected the contribution of each variable to these multivariate associations. The statistical significance of the associations was assessed via permutation testing (2000 permutations), and the robustness of the saliences was evaluated through bootstrapping (500 resamples with replacement). We interpreted the saliences (i.e., PLS correlation weights) as reflecting each variable's contribution to the multivariate association between morphometric variables and CPP-related biopsychosocial characteristics. Since the data were standardized, the saliences could be interpreted similarly to correlation coefficients. For the PLSC, residuals were used for those variables where covariates of no interest had been regressed out. Specifically, SBM metrics were adjusted as mentioned in Section 2.2.1. Alpha-amylase and area under the curve with respect to the increase of the cortisol awakening response (CAR AUC_I_) were adjusted for age, sex, contraceptive use, menstrual cycle phase, and smoking, as these are confounding factors of α-amylase and cortisol concentration (Stalder et al., 2016). Peg algometry scores were adjusted for age, sex, psychotropic medication, and non-opioid analgesics use, as these factors can confound measures of pain sensitivity (Egloff et al., 2011). Three CPP patients were excluded from analyses involving CAR AUC_I_ values due to deviations from the saliva sampling protocol, including missing samples and/or sampling delays (Δt > 5 min).

## 3 Results

### 3.1 Clinical characteristics

Table 1 and Table 2 summarize the demographic and clinical characteristics of the 30 patients with CPP (ICD-11: MG30.0) and the 30 age- and sex-matched HCs. The mean age was 41.3 years (SD = 11.6) in the CPP group and 40.9 years (SD = 13.8) in the HC group. Compared with HCs, CPP patients showed significantly higher scores on the Childhood Trauma Questionnaire (CTQ; W = 677, *p* < 0.001) and the Perceived Stress Scale (PSS; W = 797.5, *p* < 0.001), as well as significantly lower scores on the SF-36 social functioning and physical functioning subscales (W = 47.5, *p* < 0.001; W = 10.5, *p* < 0.001, respectively). In addition, CAR AUC_I_ (W = 540, *p* = 0.032), and peg algometry scores (i.e., pain sensitivity; t(49.10) = 3.93, *p* < 0.001) were increased in CPP compared with HCs. Mean α-amylase concentrations did not differ significantly between groups (W = 490, *p* = 0.559). For the description of pain sites in patients with CPP, see Supplementary Material B.5.

**Table 1:** Demographic of patients with CPP and HCs.

	CPP (N = 30)	HC (*N* = 30)	Statistics a
Age (mean, SD)	41.3 (11.6)	40.9 (13.8)	ns
Sex, females/males	24/6	24/6	ns
Smoker, yes/no	9/21	1/29	χ^2^(1) = 7.68, *p* < 0.01
Menopause, yes/no	4/20	3/21	ns
Menstrual cycle	Anovulation 6	Anovulation 5	ns
	Follicular 4	Follicular 1	
	Luteal 12	Luteal 11	
	Menstruation 2	Menstruation 3	
	Ovulation 0	Ovulation 4	
			
BDI, score	18.5 (9.8)	4.5 (3.6)	W = 851, *p* < 0.001
STAI-I, score	51.1 (11.8)	31.8 (7.0)	t(46.91) = 7.71, *p* < 0.001
STAI-II, score	53.3 (11.2)	33.9 (7.8)	t(51.70) = 7.78, *p* < 0.001
LSEQ total, score	45.2 (13.9)	52.5 (9.5)	t(51.16) = −2.35, *p* < 0.05
**Medication** (yes/no)			
Psychotropic medication	16/14	1/29	χ^2^(1) = 18.47, *p* < 0.001
Non-opioid analgesics	6/24	0/30	χ^2^(1) = 6.67, *p* < 0.01
Corticosteroids b	2/28	0/30	ns
Hormonal contraception	6/18	8/16	ns
**Biopsychological scales** (mean, SD)			
Peg algometry - mean, score e	4.79 (2.04)	3.06 (1.29)	t(49.10) = 3.93, *p* < 0.001;
			dβ = −1.13, SE = 0.54, t(54) = −2.09, *p* = 0.086
			
Peg algometry ear - mean, score e	6.90 (2.54)	4.83 (1.90)	W = 668, *p* = 0.001;
			d β = −1.37, SE = 0.71, t(54) = −1.94, *p =* 0.086
			
Peg algometry finger - mean, score e	2.68 (1.95)	1.28 (1.07)	W = 653, *p* = 0.003;
			d β = −0.88, SE = 0.50, t(54) = −1.74, *p* = 0.086
			
α-amylase [U/ml]	168.0 (90.4)	197.0 (179.0)	W = 490, ns;
			c β = 38.18, SE = 41.31, t(50) = 0.92, *p* = 0.360
CAR AUC_I_	105.0 (104.0)	47.7 (93.2)	W = 540, *p* < 0.05;
			c β = −50.83, SE = 31.03, t(47) = −1.64, *p* = 0.108
**Psychosocial scales** (mean, SD)			
CTQ total, score	50.6 (21.0)	35.2 (10.9)	W = 677, *p* < 0.001
PSS, score	24.5 (8.1)	12.7 (4.5)	W = 797.5, *p* < 0.001
SF-36 - Social functioning, score f	44.6 (24.9)	91.2 (12.8)	W = 47.5, *p* < 0.001
SF-36 - Physical functioning, score f	60.7 (23.4)	98,2 (3.8)	W = 10.5, *p* < 0.001

AUC_I_ = Area under the curve with respect to increase, BDI = Beck's Depression Inventory, CAR = Cortisol awakening response, CPP = Chronic primary pain, CTQ = Childhood Trauma Questionnaire, HC = Healthy controls, LSEQ = Leeds Sleep Evaluation Questionnaire, ns = not significant, SE = Standard Error, SD = Standard deviation, SF-36 = 36-item Short Form Health Survey, STAI-I = State-Trait Anxiety Inventory (state), STAI-II = State-Trait Anxiety Inventory (trait).

a Pearson's χ^2^ test, Fisher's Exact Test, Welch two-sample *t*-test, and Wilcoxon rank-sum test.

b Corticosteroid treatment was limited to topical administration (nasal spray) and was not administered on the day of saliva sampling.

c Group differences in α-amylase and CAR AUC_I_ were analyzed using a linear model, adjusted for age, sex, hormonal contraception, menstrual cycle, and smoking. Three patients were excluded from the CAR AUC_I_ analysis due to deviation from the saliva protocol.

d Group differences in peg algometry were analyzed using a linear model, adjusted for age, sex, psychotropic medication, and non-opioid analgesics.

e,f eScales were corrected for multiple comparisons (FDR).

**Table 2:** Clinical characteristics of patients with CPP.

	CPP (N = 30)
Symptom duration, years (mean, SD)	13.6 (12.0)
Subjective symptom load, score [0-100] (mean, SD)	50.1 (22.4)
**WPI** total, score [0-19] (mean, SD)	8.7 (3.9)
Generalized pain, cases	21
Predominantly upper-body pain, cases	6
Predominantly lower-body pain. Cases	3
**BPI intensity**, score (mean, SD)	4.6 (1.7)
Mild [0–3], cases	8
Moderate [4–6], cases	20
Severe [7–10], cases	2
**BPI interference**, score	5.6 (2.0)
Mild [0–3], cases	4
Moderate [4–6], cases	15
Severe [7–10], cases	11

BPI = Brief Pain Inventory, CPP = Chronic primary pain, SD = Standard deviation, WPI = Widespread Pain Index.

### 3.2 Structural metrics

#### 3.2.1 Cortical surface-based morphometric features

**Fig. 2:**
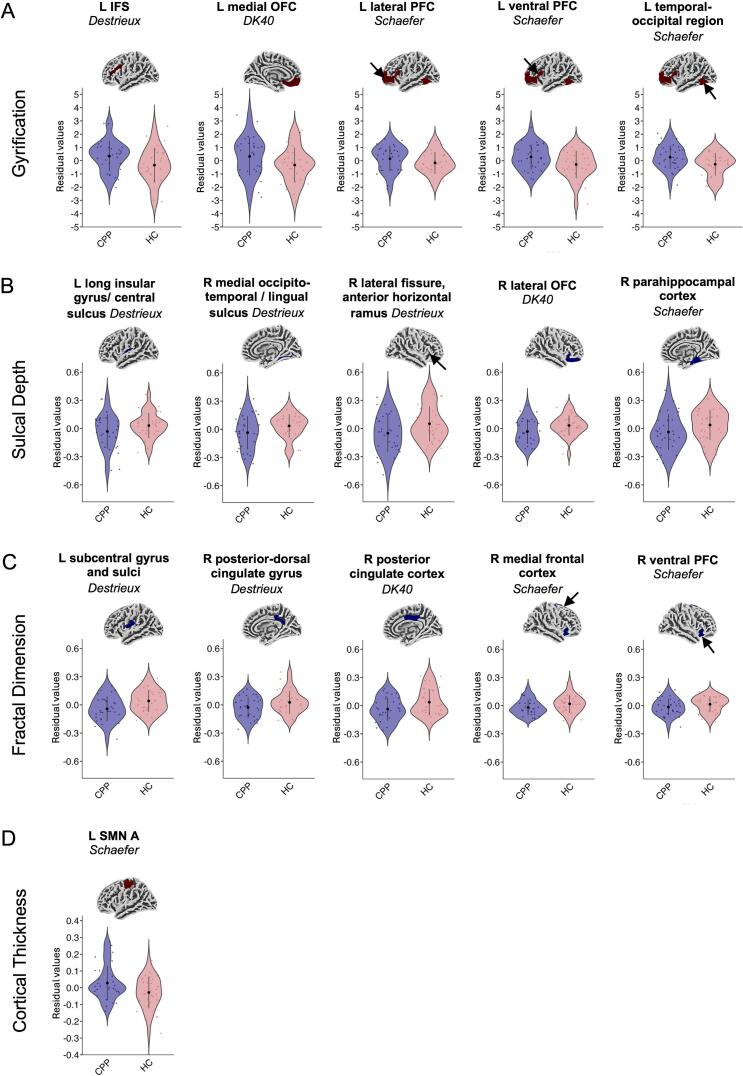
Significant ROI-wise group differences in (A) gyrification, (B) sulcal depth, and (C) fractal dimension, (D) cortical thickness in CPP patients versus HCs. Violin plots show the distribution of residual values (i.e., after covariate adjustment) for each surface metric from 30 CPP patients and 30 HCs, with mean and SD indicated, generated in R. Highlighted brain regions indicate the direction of group differences: red = CPP > HC; blue = CPP < HC, plotted in CAT12. Regions of interest (ROIs) were defined using the Destrieux and Desikan–Killiany (DK40) cortical atlases; for comparison, the Schaefer functional atlas was also included. Statistical maps were thresholded at *p* < 0.05, Holm–Bonferroni corrected for multiple comparisons across ROIs. Group differences were estimated using a general linear model (GLM) adjusted for age, sex, and a mood composite score. See Table 2 for the corresponding statistical results. IFS = Inferior frontal sulcus; L = Left; OFC = Orbitofrontal cortex; PFC = Prefrontal cortex; R = Right; SMN A = Somatomotor network A.

Compared with HCs, patients with CPP showed surface-based morphometry alterations across all three atlases. Increased gyrification in left prefrontal regions in CPP emerged as a convergent finding: it was observed in the left medial orbitofrontal cortex (DK40; *p*_HB-corr_ = 0.048), the left inferior frontal sulcus (Destrieux; *p*_HB-corr_ = 0.001), and the left lateral and ventral prefrontal cortex (Schaefer; *p*_HB-corr_ = 0.024, *p*_HB-corr_ = 0.042, respectively). Decreased sulcal depth in right lateral frontal and orbitofrontal regions in CPP showed broadly convergent or anatomically neighboring regions across atlases: it was observed in the right lateral orbitofrontal cortex (DK40; *p*_HB-corr_ = 0.046) and the right lateral fissure and anterior horizontal ramus (Destrieux; *p*_HB-corr_ = 0.03). Similarly, decreased sulcal depth in right medial temporal-occipital regions in CPP was observed in anatomically neighboring regions across atlases: it was found in the right medial occipito-temporal sulcus and lingual sulcus (Destrieux; *p*_HB-corr_ = 0.022) and the right parahippocampal cortex (Schaefer; *p*_HB-corr_ = 0.016). Additionally, decreased fractal dimension in right posterior cingulate regions in CPP converged across atlases: it was observed in the right posterior cingulate cortex (DK40; *p*_HB-corr_ = 0.045) and the right posterior-dorsal cingulate gyrus (Destrieux; *p*_HB-corr_ = 0.045); (Fig. 2, Table 3, Supplementary Material B.1).

**Table 3:** ROI-wise group differences in morphometric measures using the DK40, Destrieux, and Schaefer atlases.

	Atlas	Contrast	Region	Hemisphere	T	*p* value
**Gyrification**	DK40	CPP > HC	**Medial orbitofrontal cortex**	**left**	**2.722**	**0.048**
	Destrieux	CPP > HC	**Inferior frontal sulcus**	**left**	**3.230**	**0.001**
	Schaefer	CPP > HC	**Lateral ventral prefrontal cortex 1**	**left**	**2.025**	**0.024**
			Ventral prefrontal cortex 2	**left**	**3.368**	**0.042**
			Temporal-occipital region 1	left	3.637	0.024
**Sulcal depth**	DK40	CPP < HC	**Lateral orbitofrontal cortex**	**right**	**3.123**	**0.046**
	Destrieux	CPP < HC	Long insular gyrus and central sulcus of the insula	left	2.233	0.015
			**Lateral fissure, anterior horizontal ramus**	**right**	**3.049**	**0.030**
			**Medial occipito-temporal sulcus and lingual sulcus**	**right**	**2.712**	**0.022**
	Schaefer	CPP < HC	**Parahippocampal cortex 1**	**right**	**2.485**	**0.016**
**Fractal dimension**	DK40	CPP < HC	**Posterior cingulate cortex**	**right**	**3.290**	**0.045**
	Destrieux	CPP < HC	Subcentral gyrus and sulci	left	4.016	0.012
			**Posterior-dorsal cingulate gyrus**	**right**	**2.671**	**0.045**
	Schaefer	CPP < HC	Medial frontal cortex 1	right	2.443	0.036
			Ventral prefrontal cortex 1	right	1.862	0.034
**Thickness**	Schaefer	CPP > HC	Left somatomotor network A 1	left	3.364	0.001

The analysis included age, sex, and mood composite scores as covariates. Results were adjusted for multiple comparisons using the Holm–Bonferroni method. Bold ROIs were convergent or anatomically neighboring in at least two atlases.

Additional findings were atlas specific and were observed in patients with CPP compared to HCs. Using the Destrieux atlas, decreased sulcal depth was found in the left long insular gyrus and central sulcus of the insula (*p*_HB-corr_ = 0.015), and decreased fractal dimension in the left subcentral gyrus and sulci (*p*_HB-corr_ = 0.012). With the Schaefer atlas, increased cortical thickness was observed in the left somatomotor network A (*p*_HB-corr_ = 0.001), a gyrification increase in the left temporal-occipital region belonging to the Dorsal Attention Network A (*p*_HB-corr_ = 0.016), and decreased fractal dimension in the right medial frontal cortex of the Salience/Ventral Attention Network A and the right ventral prefrontal cortex of the Default Mode Network B (*p*_HB-corr_ = 0.036 and 0.034, respectively); (Fig. 2, Table 3, Supplementary Material B.1). A sensitivity analysis with alternative covariate adjustments is presented in Supplementary Material B.4.

Vertex-wise group comparisons of SBM metrics did not survive vertex-level FWE correction (Supplementary Fig. B.1, Table B.1).

#### 3.2.2 White matter structural connectivity (DWI)

Compared with HCs, patients with CPP showed trend-level increases in graph-theoretical centrality and connectivity (i.e., weighted degree, node degree, and eigenvector centrality) predominantly in prefrontal regions (left rostral middle and superior frontal gyri; right rostral middle and superior frontal gyri), the right postcentral gyrus, and right putamen. Conversely, CPP showed trend-level decreases in eigenvector centrality in the right isthmus cingulate, lingual, and parahippocampal gyri, as well as trend-level decreases in betweenness centrality in the bilateral cuneus and right isthmus cingulate, parahippocampal, pericalcarine, and cerebellar cortex. All analyses were adjusted for age and sex; however, none of results did not survive Benjamini–Hochberg FDR correction (see Supplementary Material B.2). Additional analyses further adjusting for mood composite score yielded no significant effects even prior to FDR correction.

### 3.3 Structural metrics associated with biopsychosocial characteristics

**Fig. 3:**
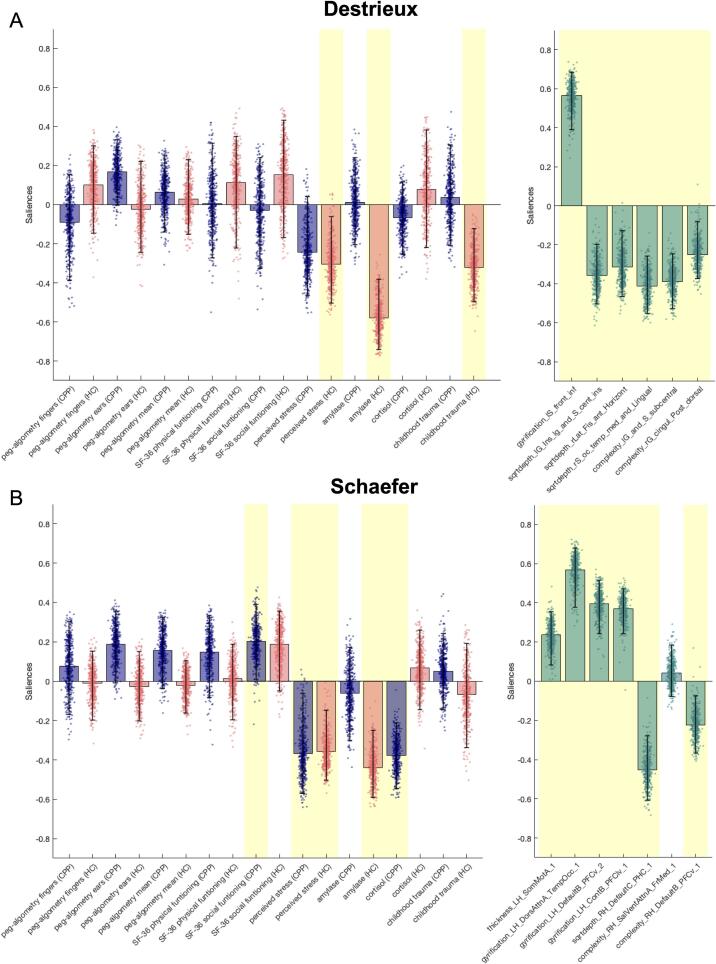
Partial Least Squares Correlation (PLSC) analysis between CPP-related biopsychosocial characteristics and surface-based morphometry (SBM) measures. The latent component (LC; optimally weighted linear combinations) for CPP-related biopsychosocial characteristics and SBM measures derived from the (A) Destrieux and (B) Schaefer atlases. Salience weights from the significant components (*p*_LC-Destrieux_ = 0.006 and *p*_LC-Schaefer_ < 0.001, respectively) indicate each variable's contribution to the multivariate pattern. Bars represent salience weights. Error bars indicate the 5th–95th percentile range of the bootstrap distribution. Yellow shading marks salience weights that were both statistically significant and robust (i.e., stable across 500 bootstrap resamples; indicated by dots). As all variables were standardized, salience weights can be interpreted similarly to correlation coefficients: variables with weights in the same direction contribute to a positive association, whereas weights in opposite directions indicate an inverse association. Childhood trauma was assessed with the Childhood Trauma Questionnaire (CTQ) and perceived stress with the Perceived Stress Scale (PSS). Amylase refers to salivary α-amylase concentration (U/mL). Cortisol refers to the cortisol awakening response (CAR), expressed as AUC_I_. Stress biomarker measures were adjusted for age, sex, hormonal contraception, menstrual cycle phase, and smoking status. Peg algometry scores were adjusted for age, sex, psychotropic medication, and non-opioid analgesics. Brain region saliences correspond to the significant regions shown in Fig. 2, and were adjusted for age, sex, and a mood composite score.

The PLSC analysis revealed significant multivariate associations between SBM features of the Destrieux atlas and CPP-related biopsychosocial factors (*p*_LC_ = 0.006, Fig. 3A, Supplementary Fig. B.4 to B.8, and Table B.6 and B.7). Positive weights of gyrification in the left inferior frontal sulcus were associated with negative weights of CTQ, PSS scores, and α-amylase in HC, but not in patients with CPP. Negative weights of sulcal depth in the left long insular gyrus, central sulcus of the insula, right lateral fissure, right anterior horizontal ramus, and the right medial occipito-temporal sulcus and lingual sulcus, and negative weights of fractal dimension in the left subcentral gyrus andsulci, and the right posterior-dorsal cingulate gyrus were also associated with these same clinical metrics in HC, but not in patients with CPP.

We then conducted the same analysis with SBM features using the Schaefer atlas. The analysis revealed significant multivariate associations between Schaefer's morphometric metrics and CPP-related biopsychosocial characteristics (*p*_LC_ < 0.001, Fig. 3B, Supplementary Fig. B.10 to B.14, and Table B.8 and B.9). The latent component showed that positive weights of cortical thickness in the left somatomotor network A and of gyrification in the left lateral and ventral prefrontal cortex and left temporal-occipital region were associated with positive weight of SF-36 social functioning in CPP, negative weights of PSS scores in HC and CPP, negative weight of α-amylase in HC, and negative weight of cortisol in CPP. Negative weights of sulcal depth in the right parahippocampal cortex and of fractal dimension in the right medial frontal cortex and the right ventral prefrontal cortex were also associated with these same clinical metrics.

In contrast, a separate PLSC analysis assessing the relationship between SBM features of the DK40 atlas and CPP-related biopsychosocial characteristics did not reveal any significant associations in patients with CPP and HC (Supplementary Fig. B.3A and B.3B). Additionally, three separate PLSC analyses assessing the relationship between SBM features across the three atlases and clinical characteristics of CPP (symptom duration, subjective symptom load, WPI total score, BPI severity score, and BPI interference score) did not reveal any significant associations (Supplementary Fig. B.3C, B.9, and B.15).

As no significant between-group differences were observed in white matter connectivity, no PLSC analysis was conducted.

## 4 Discussion

In this work, we examined whether patients with CPP differed from HCs in cortical surface architecture and white matter structural network organization and if any multimodal structural brain alterations were associated with pain- and stress-related biopsychosocial characteristics. At the level of cortical surface analyses, CPP was characterized by focal alterations in folding-related metrics: increased gyrification in the left medial orbitofrontal cortex, left lateral and ventral prefrontal cortex, and left inferior frontal regions; reduced sulcal depth in the right lateral orbitofrontal cortex, the left insula, right temporo-occipital sulci and parahippocampal cortex; and lower fractal dimension in right posterior cingulate, left subcentral territories, right medial frontal cortex and ventral prefrontal cortex. CPP was also associated with greater cortical thickness in the somatomotor cortex when compared to HCs. Several findings were robust and converged across atlases, while the rest remained atlas-specific (see Section 4.1). Multivariate correlation analyses further showed that patterns of cortical morphometry covaried with CPP-related biopsychosocial characteristics (including perceived stress, childhood trauma, and salivary biomarkers), mainly in HCs. At the level of white matter structural connectivity, no effects survived correction for multiple comparisons. Our main results suggest that CPP is associated more robustly with alterations in cortical surface morphometry than with white-matter connectivity, where we observed no significant group differences.

### 4.1 Cortical surface alterations in folding-related metrics in CPP

The present study identified focal alterations in folding-related cortical metrics (gyrification, sulcal depth, and fractal dimension) across prefrontal, insular, temporo-occipital, and cingulate regions, with additional increases in somatomotor cortical thickness in patients with CPP. The use of three parcellation schemes (DK40, Destrieux, and Schaefer) allowed us to distinguish convergent from atlas-specific findings. Several results converged across at least two atlases in overlapping or anatomically neighboring regions. Increased gyrification in left prefrontal regions and reduced sulcal depth in right lateral frontal, orbitofrontal, and temporo-occipital regions in CPP compared to HCs suggest morphometric alterations in areas implicated in top-down pain modulation, cognitive-affective pain processing, and contextual sensory integration (Kaplan et al., 2024; Vachon-Presseau et al., 2016; Zhang et al., 2024; Cascella et al., 2026). Reduced fractal dimension in right posterior cingulate areas in CPP compared to HC points to lower folding complexity in a core DMN hub, where structural differences may reflect altered self-referential pain processing and rumination, both implicated in pain chronification (Kaplan et al., 2024; Kucyi et al., 2013). These patterns are broadly consistent with prior SBM findings, notably reduced sulcal depth in frontal and insular regions in chronic shoulder pain (Niddam et al., 2019), while extending an emerging but still limited literature on gyrification, sulcal depth, and fractal dimension in CPP (Li et al., 2025b; Liu et al., 2024; Niddam et al., 2019). Sensitivity analyses indicated that results were broadly stable under additional covariate adjustment (psychotropic medication), while the reduced model (adjusting for age and sex only, omitting mood) yielded more divergent findings. This suggests that mood composite adjustment meaningfully influences the observed structural patterns in CPP, which is consistent with the known overlap between affective symptomatology and brain structure (Yu et al., 2025; Renz et al., 2025; Gómez Penedo et al., 2020; Ma et al., 2022)**,** suggesting that mood-related variance contributes in part to the observed structural patterns in CPP.

Regarding parcellation schemes, some discrepancies between the ROI-wise results derived from DK40, Destrieux, and Schaefer parcellations were expected, given differences in granularity and sulcal boundary definitions across those (Desikan et al., 2006; Destrieux et al., 2010; Schaefer et al., 2018). Atlas-specific findings, such as decreased insular sulcal depth (Destrieux) or increased somatomotor cortical thickness (Schaefer), should be interpreted carefully, as they may reflect an increased risk of false positives. Further, the absence of significant vertex-wise findings despite significant ROI-wise results likely reflects differences in statistical sensitivity between approaches: ROI-wise analyses average signal across parcels, improving signal-to-noise ratio, while vertex-wise analyses involve far more tests and consequently stricter statistical correction (Gaser et al., 2024).

Collectively, these observations suggest that the most robust structural differences in CPP converge across atlases, while atlas-specific findings and the vertex-wise null results reflect the inherent sensitivity of SBM analyses to parcellation granularity and statistical thresholding.

In contrast to the cortical surface findings, white matter structural connectivity metrics showed only trend-level group differences in CPP. Although these effects did not survive multiple-comparison correction and were attenuated after adjustment for mood, the pattern of higher FA-weighted node strength and related graph metrics in frontal and postcentral regions is broadly consistent with previous structural studies in CPP. Prior research has reported higher node strength (weighted degree) and eigenvector centrality, alongside lower betweenness centrality, across multiple regions in patients with CPP relative to HCs (Silvestro et al., 2021; Kurokawa et al., 2021; Wada et al., 2017; Pijnenburg et al., 2016). Much of the existing white matter connectivity literature in CPP has focused on primary headache and orofacial pain conditions (Silvestro et al., 2021; Kurokawa et al., 2021; Wada et al., 2017; Mei et al., 2024; Liu et al., 2017; Matoso et al., 2024), with fewer studies examining other CPP conditions (Huang et al., 2020; Pijnenburg et al., 2016). Larger multimodal studies across CPP conditions are therefore needed to determine whether consistent white matter alterations can be identified.

### 4.2 Cortical morphometry covaries with biopsychosocial dimensions

Our PLSC analyses revealed significant latent covariation patterns between distributed SBM features and CPP-related biopsychosocial characteristics, particularly stress-related measures, predominantly in HCs. We did not observe significant associations between SBM features and CPP-specific clinical characteristics such as symptom duration, symptom load, or pain severity/interference, suggesting that the observed multivariate patterns may be more closely related to stress- and adversity-linked dimensions than to pain burden itself. The significant covariation between cortical morphometry and stress-related biopsychosocial characteristics was observed mainly in HCs rather than in CPP patients. A possible interpretation is that in HCs, stress-related psychosocial and biological factors covary with cortical morphology along a continuum that is disrupted or reorganized in the context of CPP, suggesting that the normative relationship between stress systems and cortical architecture may be altered in this condition. Alternatively, greater heterogeneity within the CPP group, both in morphometric profiles and biopsychosocial characteristics, may attenuate multivariate associations relative to the more homogeneous HC group. The differential pattern of covariation across atlases further suggests that these associations are regionally specific and sensitive to parcellation resolution, consistent with the atlas-level findings discussed above. The null PLSC result for DK40 may reflect its coarser resolution, which collapses sulcal and gyral subregions that are kept distinct in Destrieux, and functionally distinct in the Schaefer, reducing sensitivity to the regionally specific covariation patterns observed in those atlases.

Although highly speculative, these findings could also be interpreted within a diathesis–stress framework (Bevers et al., 2016), suggesting a shared latent covariation pattern between stress-related biopsychosocial characteristics and cortical morphometric features. We could hypothesize that the observed morphometric profile may reflect a mixture of predispositional vulnerability, potentially linked to early adversity, and experience-dependent features shaped by more proximal stress processes. Such vulnerability-related cortical features may not be sufficient for clinical symptom expression on their own but may require additional psychosocial or biological stress to manifest. However, longitudinal studies combining repeated structural MRI, stress biomarker assessments, and psychosocial measures in individuals at risk of pain chronification are needed to test this hypothesis.

### 4.3 Limitations

The sample size in this study was modest, and the CPP group was heterogeneous with respect to the number of affected pain sites and subcategories of CPP. In addition, some participants were taking psychotropic medication and/or presented with psychiatric comorbidities. A principal diagnosis of CPP was given by clinicians, and symptoms of potential psychiatric comorbidity were assessed only using self-report questionnaires. Although symptoms of anxiety and depression were accounted for in the analysis, residual effects on brain structure cannot be entirely excluded. Moreover, due to the cross-sectional design, no causal inferences could be drawn. Finally, as in prior studies, the comparison group consisted of HCs, which does not inform whether the observed effects were specific to CPP, nor whether those could generalize to patients with chronic secondary pain.

Methodologically, direct comparison with prior DWI studies in CPP is complicated by phenotypic heterogeneity across study samples and subtle differences in acquisition, preprocessing, and modeling choices. Surface-based morphometry estimates can be sensitive to analytical choices (e.g., smoothing kernel size, topology correction, surface registration/inflation procedures, parcellation schemes, and vertex-wise versus ROI approaches), which may influence spatial specificity and statistical sensitivity. Furthermore, the relatively modest sample size in relation to the number of structural measurements increases the risk of unreliable estimates despite the use of statistical correction procedures; all findings should therefore be interpreted with appropriate caution, particularly structural connectivity findings, which did not survive statistical correction or adjustment for mood composite score. Future studies should also examine whether subcortical volumetric alterations accompany the cortical morphometric differences reported here as these structures are also highly relevant to pain processing.

## 5 Conclusion

Patients with chronic primary pain showed focal alterations in cortical surface architecture, affecting folding-related metrics in prefrontal, insular, temporo-occipital/parahippocampal, and cingulate regions, and greater somatomotor cortical thickness, whereas white matter connectivity differences remained at trend-level. Multivariate analyses indicated that biopsychosocial characteristics, specifically stress-related, covaried with distributed cortical surface patterns, predominantly in HCs. The results indicate that cortical surface morphology may be altered in patients with CPP, motivating longitudinal studies to clarify the temporal role of stress-system markers in underlying brain–pain relationships.

## Declaration of generative AI and AI-assisted technologies in the manuscript preparation process

During the preparation of this work, the author(s) used ChatGPT-5.1 in order to improve the readability and language of the manuscript. After using this tool/service, the author(s) reviewed and edited the content as needed and take(s) full responsibility for the content of the published article.

## CRediT authorship contribution statement

**Salome Häuselmann:** Writing – review & editing, Writing – original draft, Visualization, Validation, Software, Project administration, Methodology, Investigation, Formal analysis, Data curation, Conceptualization. **Anna Wyss:** Project administration, Investigation, Data curation. **Nina Bischoff:** Resources. **Rupert Bruckmaier:** Resources. **Josef Gross:** Resources. **Chantal Berna:** Writing – review & editing, Supervision, Conceptualization. **Martin grosse Holtforth:** Writing – review & editing, Supervision, Conceptualization. **Selma Aybek:** Writing – review & editing, Supervision, Resources, Funding acquisition, Conceptualization. **Nicolas Gninenko:** Writing – review & editing, Validation, Supervision, Software, Resources, Methodology, Formal analysis, Conceptualization.

## Funding

This work was supported by the 10.13039/501100001711Swiss National Science Foundation (SNF Grant PP00P3_176985 for S.A.).

## Declaration of competing interest

The authors declare that they have no known competing financial interests or personal relationships that could have appeared to influence the work reported in this paper.

## Data Availability

Data will be made available on request.
