# Supplementary material for: Structural brain alterations in chronic primary pain: a multimodal MRI study
Source: Neuroimage Clin. 2026 Jun 19;51:104023. doi: 10.1016/j.nicl.2026.104023 (PMC13320504; doi:10.1016/j.nicl.2026.104023)
Supplement: Supplementary file 1 — Supplementary material [file mmc1.docx]

SUPPLEMENTARY MATERIAL:

Structural Brain Alterations in Chronic Primary Pain: A Multimodal MRI Study

Salome Häuselmann ^1,2,3^, Anna Wyss ^1,3^, Nina Bischoff ^1^, Rupert Bruckmaier ^4^, Josef Gross ^4^, Chantal Berna ^5^, Martin grosse Holtforth ^1,6^, Selma Aybek ^7^, Nicolas Gninenko ^7^

**Author affiliations:**

1 Psychosomatic Medicine, Department of Neurology, Inselspital, Bern University Hospital, University of Bern, Switzerland

2 Graduate School of Cellular and Biomedical Sciences (GCB), University of Bern, Switzerland

3 Translational Imaging Center (TIC), Swiss Institute for Translational and Entrepreneurial Medicine, Bern, Switzerland

4 Veterinary Physiology, Vetsuisse Faculty, University of Bern, Switzerland

5 Center for Integrative and Complementary Medicine, Department of Anesthesiology, Lausanne University Hospital, Switzerland

6 Institute of Psychology, University of Bern, Switzerland

7 Department of Neurology, Faculty of Science and Medicine, University of Fribourg, Switzerland

**A. Supplementary Methods**

A.1 Psychometric and clinical assessment

A.2 Salivary cortisol and α-amylase measurements

A.3 Algometry

A.4 Diffusion-weighted imaging

**B. Supplementary Results**

B.1 Vertex-wise and ROI-wise group differences in Surface-Based Morphometry measures

B.2 Group differences in node-level structural white matter network properties in CPP patients vs. HCs

B.3 Relationship between surface-based morphometry features within the different atlases and CPP-related biopsychosocial characteristics in CPP and HC

B.4 Sensitivity analyses of group differences in ROI-wise group differences in Surface-Based Morphometry measures under alternative covariate adjustment.

B.5 Pain Distribution in CPP

**C. References**

**A. Supplementary Methods**

**A.1 Psychometric and clinical assessment**

Demographic and clinical data were collected, including age, sex, smoking status, menstrual cycle phase, menopausal status, and regular medication use. Medications were categorized as psychotropic (e.g., benzodiazepines, neuroleptics, antiepileptics, antidepressants, opioids), non-opioid analgesics (e.g., paracetamol, nonsteroidal anti-inflammatory drugs), corticosteroids, or hormonal contraceptives. Symptom duration was defined as the time between pain onset and study inclusion. On the day of MRI acquisition, subjective symptom load (visual analogue scale range: 0–100; 0=no symptoms; 100=worst symptoms experienced), anxiety, and depressive symptoms were assessed using the State–Trait Anxiety Inventory (STAI; state and trait subscales STAI-I and STAI-II, respectively)[^1^](https://www.zotero.org/google-docs/?BhKnfd) and the Beck Depression Inventory (BDI).[^2^](https://www.zotero.org/google-docs/?EWVjK8) Sleep quality during the night preceding salivary cortisol sampling was evaluated using the total Leeds Sleep Evaluation Questionnaire (LSEQ) score.[^3^](https://www.zotero.org/google-docs/?FmSdd6) Further, pain distribution over the past week was quantified with the Widespread Pain Index (WPI),[^4^](https://www.zotero.org/google-docs/?3SNGt3) which records the number of painful body regions, whereas current pain intensity (worst, least, average, and current pain within the past 24 hours) and pain-related interference across seven domains of daily functioning over the last week were assessed with the Brief Pain Inventory (BPI).[^5^](https://www.zotero.org/google-docs/?WAkCMg)

The following self-report questionnaires were obtained approximately one week before the MRI session (mean interval 6.4 ± 5.2 days): 36-item short form health survey (SF-36) for assessing the impact of symptoms on quality of life[^6^](https://www.zotero.org/google-docs/?tVg1TA), early-life stress was assessed with the 28-item Childhood Trauma Questionnaire (CTQ)[^7^](https://www.zotero.org/google-docs/?hU9OKO), which evaluates emotional, physical, and sexual abuse as well as emotional and physical neglect, whereas perceived stress during the past month was measured with the 10-item Perceived Stress Scale (PSS).[^8^](https://www.zotero.org/google-docs/?IbSGXN)

**A.2 Salivary cortisol and α-amylase measurements**

To quantify objective stress biomarkers, salivary samples were collected to determine cortisol and α-amylase concentrations as described in Häuselmann *et al*. (2026).[^9^](https://www.zotero.org/google-docs/?MkTLit) The salivary cortisol awakening response (CAR), a pronounced rise in cortisol occurring approximately 30-45 minutes after waking, is considered a sensitive index of hypothalamic–pituitary–adrenal (HPA) axis activity and endocrine stress system function.[^10–12^](https://www.zotero.org/google-docs/?T8M9Zx) In contrast, salivary α-amylase, a digestive enzyme produced by the salivary glands, is commonly used as a marker of acute stress reactivity and autonomic nervous system (ANS) activation, particularly sympathetic–adrenal–medullary (SAM) activity.[^13^](https://www.zotero.org/google-docs/?ILyPoF)

Saliva was collected using cotton swabs (Salivette Collection Devices, Sarstedt). Cortisol sampling was performed one day prior to MRI acquisition (mean 1.3 ± 1.5 days) in accordance with the recommendations by Stalder *et al*.[^12^](https://www.zotero.org/google-docs/?OQ7LgL) Participants obtained five samples across a single day: immediately upon awakening and again at 15, 30, 45, and 60 minutes after awakening. For α-amylase, saliva was collected immediately before and immediately after the MRI session.

Cortisol and α-amylase were quantified using commercially available saliva-specific enzyme immunoassays (Salimetrics; High Sensitivity Salivary Cortisol Enzyme Immunoassay kit, 1-3002; Salivary α-amylase Kinetic Enzyme Assay Kit, 1-1902) according to the manufacturers’ instructions. The CAR was operationalized as the area under the curve with respect to increase (AUC_I_), calculated from the five awakening cortisol samples.[^12,14^](https://www.zotero.org/google-docs/?LLGfOZ) For α-amylase, values from the pre- and post-MRI samples were averaged to yield a single estimate per participant.

**A.3 Algometry**

Pain sensitivity was evaluated after MRI data acquisition using a standardized and validated peg algometry pain provocation procedure (Algopeg), as described by Egloff *et al*. (2011),[^15^](https://www.zotero.org/google-docs/?sqQs5u) and Häuselmann *et al*. (2026).[^9^](https://www.zotero.org/google-docs/?YkmAyE) The procedure was conducted on both middle fingers and both earlobes. After 10 seconds of sustained clamping, participants rated their pain intensity on an 11-point numeric rating scale (NRS; 0 = no pain, 10 = worst pain imaginable). If participants could not tolerate the full 10 seconds, the trial was stopped early, and a rating of 10 was recorded. Pressure applied to the middle fingers is typically experienced at or just above the pain threshold, thereby capturing the transition from non-painful to painful sensation, whereas pressure applied to the earlobes is generally perceived as clearly suprathreshold and thus reflects pain endurance and tolerance.[^15^](https://www.zotero.org/google-docs/?UGj7SH) For statistical analyses, we computed mean values across the left and right sides for both earlobes and middle fingers, as well as an overall mean score combining finger and earlobe ratings.

**A.4 Diffusion-weighted imaging (DWI)**

**A.4.1 Anatomical Preprocessing**

Skull-stripped T1-weighted images were segmented using FreeSurfer’s ‘recon-all’ pipeline. Gray matter was parcellated into 84 brain regions (68 cortical and 16 subcortical) based on the Desikan–Killiany atlas.[^16^](https://www.zotero.org/google-docs/?GJWo3Z) All anatomical images were visually inspected to ensure data quality.

T1-weighted images were first linearly registered to DWI space using FSL’s ‘flirt’. The resulting transformation matrix was then converted to MRtrix format using MRtrix3’s ‘transformconvert’ and applied to the T1 image with MRtrix3’s ‘mrtransform’ to resample it into diffusion space. The aligned T1 image was subsequently used for 5-tissue-type (5TT) segmentation with MRtrix3’s ‘5ttgen’.

**A.4.2 DWI Preprocessing**

DWI data were processed using MRtrix3, following an adapted version of the Human Connectome Project preprocessing pipeline.[^17–19^](https://www.zotero.org/google-docs/?UlOln5) Steps included brain masking, Marchenko–Pastur principal component analysis (MP-PCA) denoising, Gibbs ringing artefacts removal, motion and Eddy current correction, intensity normalization, and coregistration to the T1-weighted images using FSL’s ‘flirt’. Susceptibility-induced distortion correction was not applied due to the absence of reverse phase-encoded b_0_ images.

**A.4.3 Structural Connectome Reconstruction**

To reconstruct the structural connectome, probabilistic anatomically constrained tractography (ACT) was performed using MRtrix3’s ‘tckgen’ tool with parameters set to generate 100 million streamlines, a maximum fiber length of 250 mm, and a cutoff value of 0.06. Seeding was performed dynamically within ‘tckgen’ using the generated fiber orientation distribution (FOD) map rather than a predefined seed image, ensuring that the seeding density reflected the local white matter content for whole-brain tractography. Specifically, this FOD map was first computed from the preprocessed diffusion data using ‘dwi2fod’ within MRtrix3 and subsequently used in ‘tckgen’.

The resulting tractogram was filtered to 10% (10 million streamlines) using MRtrix3’s spherical-deconvolution informed filtering of tractograms (SIFT) algorithm[^20^](https://www.zotero.org/google-docs/?2uGplA) ‘tcksift’ to improve quantitative accuracy by reducing reconstruction bias. From the filtered tractogram, 84 × 84 structural connectomes were generated using MRtrix3’s ‘tck2connectome’ tool, based on the previously obtained Desikan–Killiany atlas parcellation. For each connectome, we extracted the mean fractional anisotropy (FA) values. See Gninenko *et al*. (2025)[^17^](https://www.zotero.org/google-docs/?x05PNx) for more details. Five HCs and one patient with CPP had to be excluded from the analysis due to corrupted DWI files.

**A.4.4 Node-level Graph-Theoretical Analysis**

To characterize node-level features of structural white matter network organization across the 84 regions defined by the Desikan–Killiany atlas, we computed node-level graph-theoretical metrics from each participant’s normalized FA-weighted connectome. Weighted degree (WD) was computed directly from the FA-weighted connectome according to its standard definition, whereas node degree, local clustering coefficient, local efficiency, eigenvector centrality, and betweenness-based metrics were computed in MATLAB using functions from the Brain Connectivity Toolbox (BCT).[^21^](https://www.zotero.org/google-docs/?sceFKA) For the connection-lengths matrix metric, namely betweenness centrality, we converted the FA-weighted adjacency matrix to an edge-length matrix using the BCT (i.e, weight_conversion(·, 'lengths')), such that higher FA weights corresponded to shorter path lengths.

*(i) Weighted degree (WD)*

We computed the weighted degree (node strength) for each region, defined as the sum of the weights of all edges incident to node *i*:

${WD}_{i}=\sum_{j=1}^{N} w_{i,j}$ ,

where N=84 is the total number of regions and w_ij_ denotes the FA-based weight of the connection between regions *i* and *j*. For each participant, WD values were stored as a 1×84 vector, with entry *i* representing the cumulative strength of all weighted connections of region *i* to all other regions.

*(ii) Node Degree*

Node degree quantifies the number of links connected to an individual node.[^21^](https://www.zotero.org/google-docs/?OhbMJ1)

(iii) Local Clustering Coefficient

The local clustering coefficient is the average “intensity” (geometric mean) of all triangles associated with an individual node.[^21^](https://www.zotero.org/google-docs/?CDjtep)

*(iv) Local Efficiency*

Local efficiency quantifies the efficiency of information transfer within the immediate neighborhood of a node, computed as the global efficiency of the subgraph induced by its first-order neighbors, and reflects the extent to which neighboring nodes remain well connected via short paths independent of the node itself.[^21^](https://www.zotero.org/google-docs/?ybbbJ0)

*(v) Eigenvector centrality*

Eigenvector centrality was computed to capture the extent to which a node is connected to other highly connected nodes. Specifically, eigenvector centrality assigns higher values to nodes that are connected to nodes with high centrality.[^21^](https://www.zotero.org/google-docs/?rrMeuV)

*(vi) Node betweenness centrality*

To quantify the importance of regions for network-wide communication, we computed node betweenness centrality, defined as the fraction of all shortest paths in the network that pass through a given node. Nodes with high betweenness centrality participate in a large proportion of shortest paths and can be interpreted as critical hubs for information flow.[^21^](https://www.zotero.org/google-docs/?1zjcu2)

**A.4.5 Statistical analysis for DWI**

Group differences in node-level features of structural white matter network organization between HC (N = 25) and patients with CPP (N = 29) were assessed separately for each ROI using general linear models (GLMs). For each node-level metric, we fit ROI-wise linear models with Group (CPP vs. HC) as the predictor of interest and age and sex as covariates. To control for multiple comparisons across the 84 regions, Group-effect *p*-values were adjusted using the Benjamini–Hochberg false discovery rate (FDR) procedure with *q* = 0.05. Hedges’ *g* effect sizes for the group contrast were derived from the ROI-wise GLMs by standardizing the adjusted group coefficient (β_CPP–HC_) with the model residual standard deviation and applying the small-sample correction factor J (yielding Hedges’ *g*).[^22^](https://www.zotero.org/google-docs/?KK0kNC)

**B. Supplementary Results**

**B.1 Vertex-wise and ROI-wise group differences in Surface-Based Morphometry measures**

In CPP patients compared with HCs, vertex-wise group comparisons showed increased cortical thickness in the left precentral gyrus (DK40) and, correspondingly, the left central sulcus (Destrieux) (*p* = 0.009), as well as decreased sulcal depth in the right insula (DK40) and, correspondingly, the right anterior circular sulcus of the insula (Destrieux) (*p* = 0.019) (Supplementary Fig. 1 and Supplementary Table 1). No effects survived vertex-wise FWE correction (p_FWE_ < 0.05).


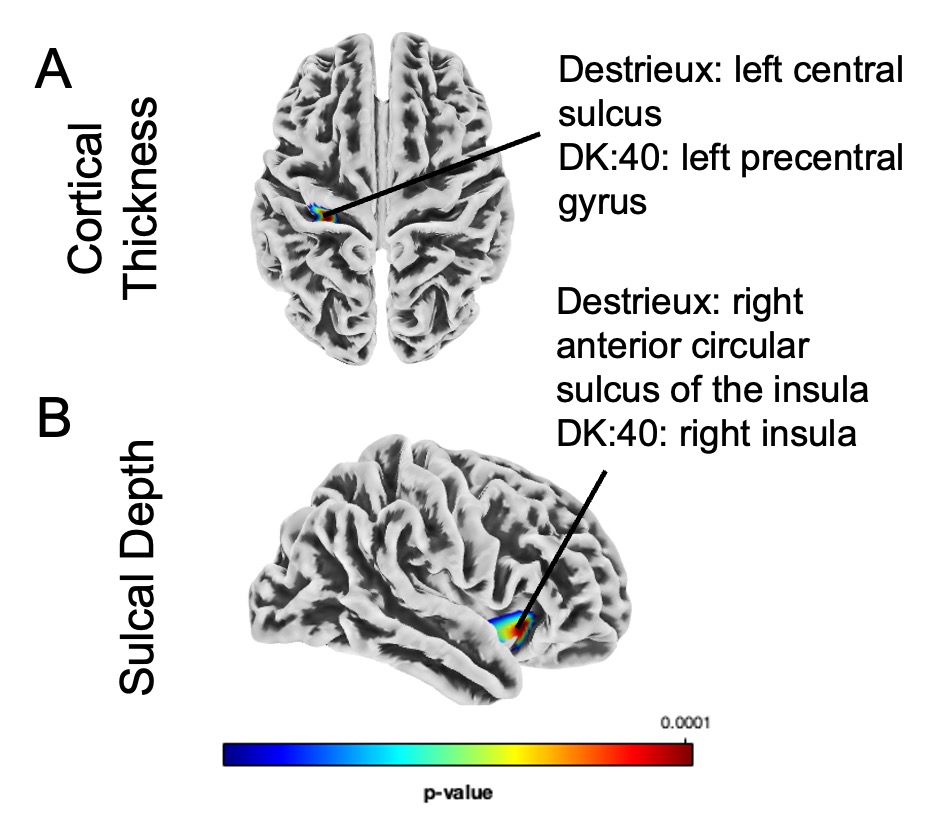


**Supplementary Fig. B.1: Vertex-wise group differences in (A) cortical thickness (CPP > HC) and (B) sulcal depth (CPP < HC) displayed on the cortical surface.** Maps were thresholded using a cluster-forming threshold of vertex-wise p < 0.001 (uncorrected) with a minimum cluster extent of k ≥ 20 vertices. Cluster-level inference was performed using FWE correction (p_FWE_ < 0.05). No effects survived vertex-wise FWE correction (p_FWE_ < 0.05). Analyses were adjusted for age, sex, and a mood composite score.

**Supplementary Table B.1: Group differences in morphometric parameters on a vertex level.**

|  |  |  |  |  | **peak-level** | **cluster-level** | |
| --- | --- | --- | --- | --- | --- | --- | --- |
|  | **Contrast** | **Region Destrieux** | **Region DK40** | **MNI-Coordinates** | **T** | **p_FWE-corr_** | **k_E_** |
| **Cortical thickness** | CPP>HC  CPP<HC | **Left central sulcus**  Right precuneus gyrus | **Left precentral gyrus**  Right precuneus | **[-28 -29 52]**  [14 -73 41] | **4.00**  3.91 | **0.009***  0.152 | **188**  84 |
| **Gyrification** | CPP>HC | Left Inferior frontal gyrus, triangular part  Left occipito- temporal gyrus, anterior part  Left inferior frontal sulcus  Left superior occipital gyrus | Left inferior frontal gyrus, pars triangularis  Left fusiform gyrus  Left caudal middle frontal gyrus  Left superior parietal lobule | [-48 24 5]  [-41 -67 -10]  [-37 15 30]  [-17 -87 32] | 3.96  3.57  3.53  3.41 | 0.188  0.404  0.451  0.582 | 78  43  37  23 |
| **Sulcal depth** | CPP<HC | **Right anterior circular sulcus of the insula**  Left middle frontal sulcus  Left subcentral gyrus and sulci | **Right insular cortex**  Left rostral middle frontal gyrus  Left postcentral gyrus | **[30 20 -7]**  [-25 50 3]  [-28 46 12]  [-63 -8 24] | **3.71**  3.52  3.46  3.44 | **0.019***  0.169  0.570 | **185**  87  22 |
| **Fractal dimension** | CPP>HC  CPP<HC | Right inferior temporal sulcus  Right occipital pole  Left subcentral gyrus and sulci  Left precentral gyrus | Right middle temporal gyrus  Right lingual gyrus  Left postcentral gyrus  Left precentral gyrus | [52 -57 -1]  [8 -94 -5]  [-62 -10 9]  [-18 -23 73] | 3.91  3.55  3.77  3.52 | 0.533  0.483  0.089  0.616 | 29  34  102  21 |

The analysis included age, sex, and mood composite scores as covariates. Results are reported at an uncorrected vertex-wise threshold of *p* < 0.001. While no clusters survived vertex-level family-wise error (FWE) correction, the table lists clusters significant at the cluster-level FWE-corrected threshold with a cluster extent of k ≥ 20 vertices; these clusters are marked in bold and with an asterisk (*). Regions are labelled according to the Desikan–Killiany atlas. kE, cluster extent.

**Supplementary Table B.2: ROI-wise group differences in morphometric measures using the DK40 atlas.**

|  | **Contrast** | **Region** | **Hemisphere** | **T** | **p-value** |
| --- | --- | --- | --- | --- | --- |
| **Cortical thickness** | CPP>HC | Postcentral gyrus  Pericalcarine cortex  Paracentral lobule  Pars orbitalis of the inferior frontal gyrus | left  left  right  right | 2.092488  1.768937  1.870069  1.711587 | 0.020511  0.041225  0.033400  0.046304 |
| **Gyrification** | CPP>HC  CPP<HC | Lateral orbitofrontal cortex  Rostral middle frontal gyrus  Pars opercularis of the inferior frontal gyrus  Inferior temporal gyrus  Pars orbitalis of the inferior frontal gyrus  Pars triangularis of the inferior frontal gyrus  Caudal middle frontal gyrus  Lateral occipital cortex  **Medial orbitofrontal cortex**  Triangular part of the inferior frontal gyrus  Lateral occipital cortex  Isthmus of the cingulate cortex  Transverse temporal cortex | left  left  left  left  left  left  left  left  **left**  right  right  right  right | 2.944151  2.519733  2.371226  2.232040  2.220793  2.108685  2.077403  2.035254  **2.722170**  2.714024  1.854050  2.536020  2.220852 | 0.002368  0.007339  0.010629  0.014853  0.015251  0.019770  0.021223  0.023328  **0.047686*** 0.004430  0.034549  0.007041  0.015249 |
| **Sulcal depth** | CPP>HC  CPP<HC | Pericalcarine cortex  Insular cortex  Rostral middle frontal gyrus  Superior frontal gyrus  Postcentral gyrus  Lateral orbitofrontal cortex  Paracentral lobule  Insular cortex  Pars triangularis of the inferior frontal gyrus  Temporal pole  Parahippocampal gyrus  Fusiform gyrus  Rostral middle frontal gyrus  Transverse temporal cortex  Middle temporal gyrus  Cuneus  **Lateral orbitofrontal cortex** | right  left  left  left  left  left  left  right  right  right  right  right  right  right  right  right  **right** | 2.056967  2.543923  2.435121  2.417569  2.138742  2.021661  1.980586  2.900382  2.713715  2.531488  2.316024  2.226880  1.952420  1.810346  1.722059  1.684560  **3.123317** | 0.022222  0.006901  0.009079  0.009483  0.018456  0.024044  0.026324  0.002674  0.004434  0.007123  0.012156  0.015034  0.027993  0.037853  0.045339  0.048870  **0.045622*** |
| **Fractal dimension** | CPP>HC  CPP<HC | Transverse temporal cortex  Pericalcarine cortex  Caudal anterior cingulate cortex  Temporal pole  Parahippocampal gyrus  Frontal pole  **Posterior cingulate cortex** | right  right  left  left  right  right  **right** | 1.831962  1.778171  2.320139  1.710233  2.608341  1.779861  **3.290052** | 0.036187  0.040452  0.012035  0.046429  0.005846  0.040312  **0.044667*** |

The analysis included age, sex, and mood composite scores as covariates. P-values were adjusted for multiple comparisons using the Holm–Bonferroni method. Holm–Bonferroni–corrected *p*-values < 0.05 were considered statistically significant and are shown in bold and marked with an asterisk (*).

**Supplementary Table B.3: ROI-wise group differences in morphometric measures using the Destrieux atlas.**

|  | **Contrast** | **Region** | **Hemisphere** | **T** | **p-value** |
| --- | --- | --- | --- | --- | --- |
| **Cortical thickness** | CPP>HC  CPP<HC | Central sulcus  Postcentral gyrus  Lateral fissure, anterior horizontal ramus  Calcarine sulcus  Calcarine sulcus  Inferior circular sulcus of the insula  Central sulcus  Medial orbital sulcus and olfactory sulcus  Inferior occipital gyrus and sulcus  Lingual gyrus (medial occipito-temporal)  Precuneus gyrus  Posterior transverse collateral sulcus | left  left  left  left  right  right  right  right  right  right  right  right | 2.664635  2.407913  1.768211  1.746197  2.121905  1.871088  1.781555  2.149353  2.044079  1.848973  1.727530  1.695493 | 0.005047  0.009712  0.041286  0.043180  0.019183  0.033328  0.040172  0.018011  0.022873  0.034920  0.044842  0.047818 |
| **Gyrification** | CPP>HC  CPP<HC | **Inferior frontal sulcus**  H-shaped orbital sulcus  Superior occipital gyrus  Medial occipito-temporal and lingual sulci  Inferior occipital gyrus and sulcus  Lateral occipito-temporal sulcus  Lateral fissure, anterior vertical ramus  Precentral sulcus, inferior part  Orbital gyrus  Gyrus rectus  Inferior temporal gyrus  Inferior frontal gyrus, triangular part  Sulcus intermedius primus (Jensen sulcus)  Middle occipital gyrus  Precentral gyrus  Lateral orbital sulcus  Middle-anterior cingulate gyrus and sulcus  Parieto-occipital sulcus  Inferior frontal gyrus, triangular part  Lateral orbital sulcus  Transverse temporal gyrus  Pericallosal sulcus  Transverse temporal gyrus  Paracentral lobule and sulcus  Posterior-ventral cingulate gyrus | **left**  left  left  left  left  left  left  left  left  left  left  left  left  left  left  left  right  right  right  right  left  right  right  right  right | **3.230054**  3.200843  2.770953  2.725244  2.720607  2.710902  2.646834  2.421280  2.315460  2.271631  2.240852  2.171416  2.143884  2.049948  1.975760  1.802283  2.364853  2.133126  1.806345  1.792237  1.989288  2.770107  2.273221  2.020141  1.917835 | **0.001045***  0.001139  0.003805  0.004300  0.004353  0.004467  0.005288  0.009396  0.012172  0.013521  0.014547  0.017115  0.018239  0.022574  0.026604  0.038491  0.010796  0.018696  0.038168  0.039298  0.003814  0.013470  0.013470  0.024125  0.030165 |
| **Sulcal depth** | CPP>HC  CPP<HC | Precuneus  Short insular gyri  Middle frontal sulcus  Frontomarginal gyrus and sulcus  Subcentral gyrus and sulci  Medial orbital sulcus and olfactory sulcus  **Long insular gyrus and central sulcus of the insula**  Superior circular sulcus of the insula  Superior frontal gyrus  Inferior circular sulcus of the insula  Anterior circular sulcus of the insula  Central sulcus  Paracentral lobule and sulcus  H-shaped orbital sulcus  Anterior circular sulcus of the insula  **Lateral fissure, anterior horizontal ramus**  Long insular gyrus and central sulcus of the insula  Inferior circular sulcus of the insula  **Medial occipito-temporal sulcus and lingual sulcus**  Planum polare (superior temporal gyrus, anterior)  Short insular gyri  Temporal pole  Parahippocampal gyrus (medial occipito-temporal gyrus, parahippocampal part)  Medial orbital sulcus and olfactory sulcus  Lateral fissure, anterior vertical ramus  Superior circular sulcus of the insula  Frontomarginal gyrus and sulcus  Lateral fissure, posterior ramus  Orbital gyrus  H-shaped orbital sulcus  Superior frontal gyrus  Inferior frontal gyrus, triangular part  Middle temporal gyrus  Parieto-occipital sulcus  Middle frontal gyrus  Transverse temporal sulcus | right  left  left  left  left  left  **left**  left  left  left  left  left  left  left  right  **right**  right  right  **right**  right  right  right  right  right  right  right  right  right  right  right  right  right  right  right  right  right | 1.684741  3.066968  2.963029  2.829022  2.801585  2.343448  **2.233260**  2.178173  2.118727  1.906735  1.899901  1.752353  1.728573  1.708035  3.206696  **3.049318**  2.862265  2.746484  **2.712176**  2.579844  2.543169  2.387710  2.330541  2.204899  2.200304  2.197923  2.143946  2.126872  2.122720  2.116192  2.103849  1.958271  1.754907  1.715441  1.708076  1.700955 | 0.048853  0.001676  0.002247  0.003252  0.003503  0.011375  **0.014810***  0.016849  0.019322  0.030892  0.031347  0.042643  0.044748  0.046634  0.001119  **0.029951***  0.002970  0.004063  **0.022259***  0.006293  0.006914  0.010208  0.011736  0.015831  0.016002  0.016091  0.018237  0.018966  0.019147  0.019435  0.019989  0.027639  0.042422  0.045947  0.046631  0.047300 |
| **Fractal dimension** | CPP>HC  CPP<HC | Transverse temporal gyrus (Heschl’s gyrus)  Frontomarginal gyrus and sulcus  Occipital pole  Long insular gyrus and central sulcus of the insula  Medial orbital sulcus and olfactory sulcus  Anterior transverse collateral sulcus  **Subcentral gyrus and sulci**  Middle temporal gyrus  Planum polare (superior temporal gyrus, anterior)  Planum temporale (superior temporal gyrus, posterior)  Middle-anterior cingulate gyrus and sulcus  Inferior occipital gyrus and sulcus  **Posterior-dorsal cingulate gyrus**  Parahippocampal gyrus (medial occipito-temporal gyrus, parahippocampal part)  H-shaped orbital sulcus  Middle-posterior cingulate gyrus and sulcus  Subcentral gyrus and sulci  Middle-anterior cingulate gyrus and sulcus  Inferior frontal gyrus, orbital part  Superior occipital gyrus  Transverse frontopolar gyri and sulci | right  right  right  right  right  right  **left**  left  left  left  left  left  **right**  right  right  right  right  right  right  right  right | 2.333305  2.326779  0.034234  1.773332  1.769733  1.681313  **4.015880**  2.395477  2.286360  1.987873  1.923692  1.794611  **2.670771**  2.567042  2.234733  2.221252  2.128844  2.010659  1.796660  1.717024  1.696590 | 0.011658  0.011844  0.034234  0.040855  0.041157  0.049187  **0.012407***  0.001015  0.013054  0.025906  0.029787  0.039106  **0.044699***  0.006504  0.014758  0.015235  0.018880  0.024637  0.038941  0.045801  0.047714 |

The analysis included age, sex, and mood composite scores as covariates. P-values were adjusted for multiple comparisons using the Holm–Bonferroni method. Holm–Bonferroni–corrected *p*-values < 0.05 were considered statistically significant and are shown in bold and marked with an asterisk (*).

**Supplementary Table B.4: ROI-wise group differences in morphometric measures using the Schaefer atlas.**

|  | **Contrast** | **Region** | **Hemisphere** | **T** | ***p*-value** |
| --- | --- | --- | --- | --- | --- |
| **Cortical thickness** | CPP>HC  CPP<HC | **SMN A, R1**  Control Network C, Precuneus, R1 | **left**  right | **3.363983**  2.357136 | **0.001405***  0.011002 |
| **Gyrification** | CPP>HC  CPP<HC | **DAN A, Temporal-Occipital Cortex, R1**  **DMN B, Ventral Prefrontal Cortex, R2**  Control Network A, Lateral PFC, R1  Limbic Network B, Orbitofrontal Cortex, R1  Visual Peripheral Network, Inferior Extrastriate Cortex, R1  Visual Central Network, Extrastriate Cortex, R3  Visual Central Network, Extrastriate Cortex, R1  Control Network A, Lateral PFC, R2  **Control Network B, Lateral PFC ventral, R1**  DMN A, Medial PFC, R1  Visual Central Network, Extrastriate Cortex, R2  Salience/VAN B, Lateral PFC, R1  DMN B, Lateral PFC, R1  Salience/VAN A, Medial Frontal Cortex, R1  Visual Central Network, Extrastriate Cortex, R3  DMN B, Ventral PFC, R2  Control Network C, Posterior Cingulate Cortex, R1 | **left**  **left**  left  left  left  left  left  left  **left**  left  left  left  left  right  right  right  right | **3.636783**  **3.367768**  3.007808  2.991135  2.101179  2.091758  2.045671  2.030356  **2.025269**  1.960198  1.909414  1.849932  1.757315  1.991380  1.985170  1.949157  2.005875 | **0.001219***  **0.041678***  0.001981  0.002077  0.020111  0.020545  0.022792  0.023584  **0.023852***  0.027523  0.030715  0.034849  0.042215  0.025708  0.025708  0.025708  0.024899 |
| **Sulcal depth** | CPP<HC | Control Network B, Lateral-Ventral PFC, R1  SMN B, Secondary Somatosensory Cortex, R2  SMN B, Central Sulcus, R1  Salience/VAN A, Insula, R1  DMN B, Dorsal PFC, R1  DMN B, Ventral PFC, R1  Salience/VAN B, Lateral PFC, R1  Salience/VAN A, Insula, R2  SMN B, Secondary Somatosensory Cortex, R1  SMN A, R2  DMN B, Ventral PFC, R2  Limbic Network B, Orbitofrontal Cortex, R1  Control Network B, Lateral-Ventral PFC, R1  DMN B, Ventral PFC, R1  Limbic Network B, Orbitofrontal Cortex, R1  **DMN C, Parahippocampal Cortex, R1**  Limbic Network A, Temporal Pole, R1  Visual Peripheral Network, Sup. Extrastriate Cortex, R1  Visual Central Network, Extrastriate Cortex, R1  DMN B, Ventral PFC, R2  Salience/VAN A, Insula, R1  SMN B, Secondary Somatosensory Cortex, R1  SMN A, R3 | left  left  left  left  left  left  left  left  left  left  left  left  right  right  right  **right**  right  right  right  right  right  right  right | 2.711028 2.468583 2.270340 2.235037 2.149235 2.096046 2.083554 1.967809 1.918761 1.854356 1.772808  1.702926 2.795567 2.705298 2.553240 **2.484746** 2.367127 2.309841 2.141905  2.114957 2.110866 2.094278  1.771413 | 0.004465  0.008351  0.013563  0.014748  0.018016  0.020347  0.020931  0.027070  0.030105  0.034526  0.040899  0.047114  0.003561  0.004534  0.006738  **0.016036***  0.010737  0.012338  0.018323  0.019489  0.019672  0.020428  0.041016 |
| **Fractal dimension** | CPP>HC  CPP<HC | Visual Central Network, Striate Cortex, R1  DAN A, Parietal-Occipital Cortex, R1  Visual Peripheral Network, Striate-Calcarine Cortex, R1  DMN B, Secondary Somatosensory Cortex, R2  Salience/VAN B, Medial PFC, R1  SMN B, Secondary Somatosensory Cortex, R2  **Salience/VAN A, Frontal Medial Cortex, R1**  DMN B, Ventral PFC, R2  DMN C, Retrosplenial Cortex, R1  Control Network C, Posterior Cingulate Cortex, R1  **DMN B, Ventral PFC, R1**  DMN C, Parahippocampal Cortex, R1  Salience/VAN B, Inferior Parietal Lobule, R1 | left  right  right  left  left  right  **right**  right  right  right  **right**  right | 1.919396  2.252177 1.947563  3.229069 2.039554 2.550586 **2.442784** 1.941062 1.929184 1.865343 **1.862340**  1.749477 1.730419 | 0.030064  0.014162  0.028290  0.001048  0.023105  0.006784  **0.035628***  0.028691  0.029437  0.033735  **0.033950***  0.042893  0.044582 |

The analysis included age, sex, and mood composite scores as covariates. P-values were adjusted for multiple comparisons using the Holm–Bonferroni method. Holm–Bonferroni–corrected *p*-values < 0.05 were considered statistically significant and are shown in bold and marked with an asterisk (*). DAN = Dorsal Attention Network, DMN = Default Mode Network; PFC = Prefrontal Cortex; SMN = Somatomotor Network; R = Region; VAN = Ventral Attention Network.

**B.2 Group differences in node-level structural white matter network properties in CPP patients vs. HCs**

First, we saw increased weighted degrees in patients with CPP compared to HCs in the left rostral middle frontal gyrus, left superior frontal gyrus, right postcentral gyrus, and right superior frontal gyrus (Supplementary Fig. B.2A, Supplementary Table B.5). Second, patients with CPP compared to HCs also showed increased node degrees in the following ROIs: left rostral middle frontal gyrus, left superior frontal gyrus, right putamen, right postcentral gyrus, right rostral middle frontal gyrus, and right superior frontal gyrus (Supplementary Fig. B.2B, Supplementary Table B.5). Third, eigenvector centrality values were increased in left rostral middle frontal gyrus, left superior frontal gyrus, right postcentral gyrus, right rostral middle frontal gyrus, and right superior frontal gyrus, and eigenvector centrality values were decreased in right isthmus of the cingulate gyrus, right lingual gyrus, and right parahippocampal gyrus in patients with CPP compared to HCs (Supplementary Fig. B.2C, Supplementary Table B.5). Finally, patients with CPP compared with HCs showed decreased node betweenness centrality values in left cuneus, right cuneus, right isthmus of the cingulate gyrus, right parahippocampal gyrus, right pericalcarine cortex, and right cerebellar cortex (Supplementary Fig. B.2D, Supplementary Table B.5). Results did not survive Benjamini–Hochberg false discovery rate correction.

**Supplementary Fig. B.2: Group differences in node-level structural white matter network properties in CPP patients vs. HCs.** (A) Weighted degree, (B) node degree, (C) eigenvector centrality, (D) betweenness centrality (Desikan–Killiany atlas). Regions are shown at uncorrected ROI-wise *p* < 0.05, adjusted for age and sex. Colors indicate *t*-values (CPP–HC): positive (green) = CPP > HC; negative (yellow) = CPP < HC. No results survived Benjamini–Hochberg FDR correction.

**Supplementary Table B.5: ROI-group differences in node-level structural white matter network properties**

|  | **Region DK40** | **Hemisphere** | **ß_CPP-HC_** | **[95% CI]** | **t(df)** | **p-value** | **Hedges’ g** |
| --- | --- | --- | --- | --- | --- | --- | --- |
| **Weighted Degree** | Rostral middle frontal gyrus  Superior frontal gyrus  Postcentral gyrus  Superior frontal gyrus | left  left  right  right | 4.7327  4.8611  3.9241  5.6922 | [1.081, 8.385]  [0.139, 9.583]  [0.223,7.625]  [0.272, 11.112] | 2.6029(50)  2.0677(50)  2.1298(50)  2.1094(50) | 0.0121  0.0438  0.0381  0.0399 | 0.7012  0.5570  0.5737  0.5682 |
| **Node degree** | Rostral middle frontal gyrus  Superior frontal gyrus  Putamen  Postcentral gyrus  Rostral middle frontal gyrus  Superior frontal gyrus | left  left  right  right  right  right | 6.2565  7.1403  2.8479  5.3572  4.8677  8.2951 | [1.6718, 10.8412]  [0.8057, 13.4748]  [0.1505, 5.5453]  [0.8145, 9.8998]  [0.3243, 9.4112]  [0.8302, 15.7600] | 2.7410(50)  2.2640(50)  2.1207(50)  2.3687(50)  2.1519(50)  2.2319(50) | 0.0085  0.0279  0.0389  0.0218  0.0363  0.0301 | 0.7384  0.6099  0.5713  0.6381  0.5797  0.6013 |
| **Eigenvector centrality** | Rostral middle frontal gyrus  Superior frontal gyrus  Isthmus of the cingulate gyrus  Lingual gyrus  Parahippocampal gyrus  Postcentral gyrus  Rostral middle frontal gyrus  Superior frontal gyrus | left  left  right  right  right  right  right  right | 0.0109  0.0114  -0.0078  -0.0068  -0.0126  0.0071  0.0075  0.0136 | [0.0046, 0.0174]  [0.0018, 0.0210]  [-0.0144, -0.0012]  [-0.0135, −0.0001]  [-0.0240, -0.0012]  [0.0012,0.0130]  [0.0011, 0.0140]  [0.0021, 0.0251] | 3.4601(50)  2.3790(50)  -2.3894(50)  -2.0402(50)  -2.2184(50)  2.4139(50)  2.3415(50)  2.3812(50) | 0.0011  0.0212  0.0207  0.0466  0.0311  0.0195  0.0232  0.0211 | 0.9321  0.6409  -0.6437  -0.5496  -0.5976  0.6503  0.6308  0.6415 |
| **Node betweenness centrality** | Cuneus  Cuneus  Isthmus of the cingulate gyrus  Parahippocampal gyrus  Pericalcarine cortex  Cerebellar cortex | left  right  right  right  right  right | -11.4509  -14.1337  -18.7436  -51.3676  -8.6945  -28.1593 | [-20.5723, -2.3295]  [-25.2815,-2.9858]  [-33.2773, -4.2099]  [-4.2099, -3.8812]  [-16.0163, -1.3727]  [-54.0382, -2.2804] | -2.5215(50)  -2.5465(50)  -2.5904(50)  -2.1727(50)  -2.3851(50)  -2.1856(50) | 0.0149  0.0140  0.0125  0.0125  0.0209  0.0336 | -0.6793  -0.6860  -0.6978  -0.5853  -0.5853  -0.5888 |

The analysis included age and sex as covariates. No results survived Benjamini–Hochberg FDR correction.

**B.3 Relationship between surface-based morphometry features within the different atlases and CPP-related biopsychosocial characteristics in CPP and HC**

**B.3.1 DK40 Atlas**

A

B

**
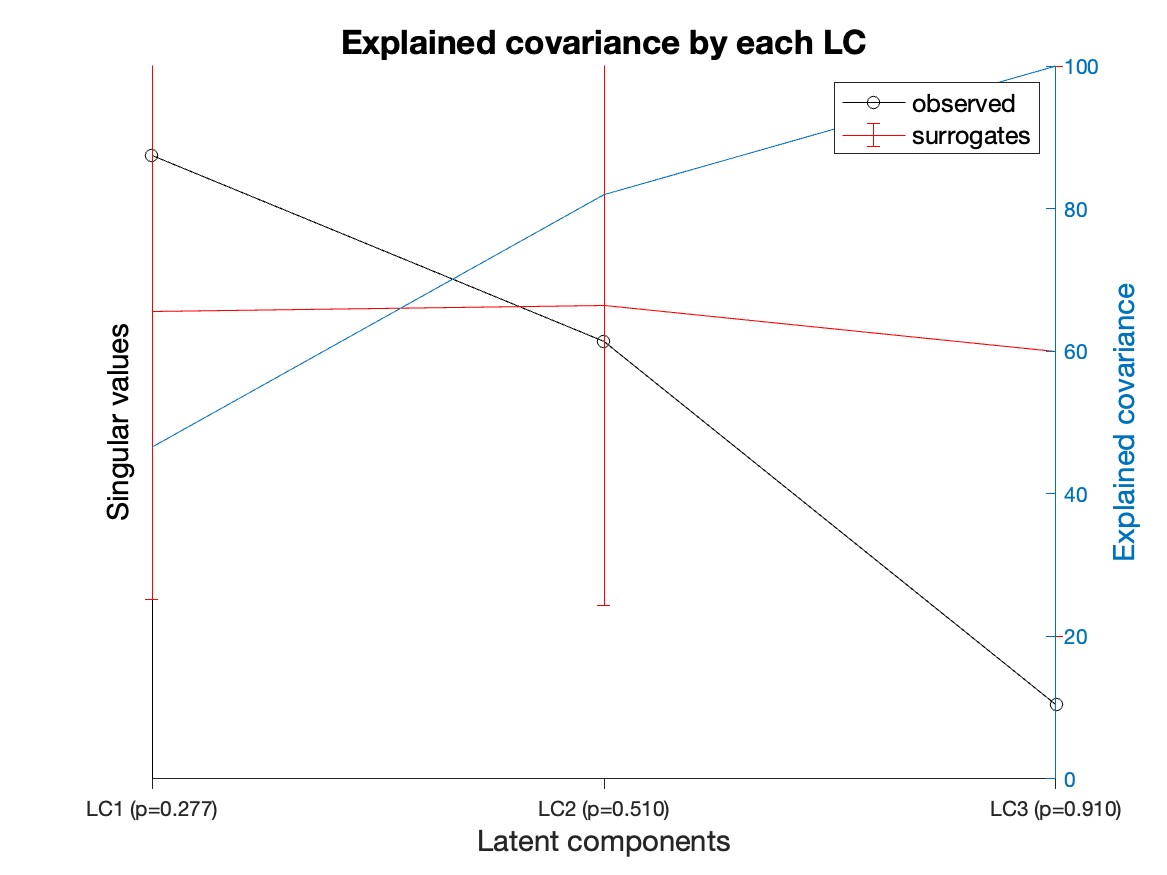
***
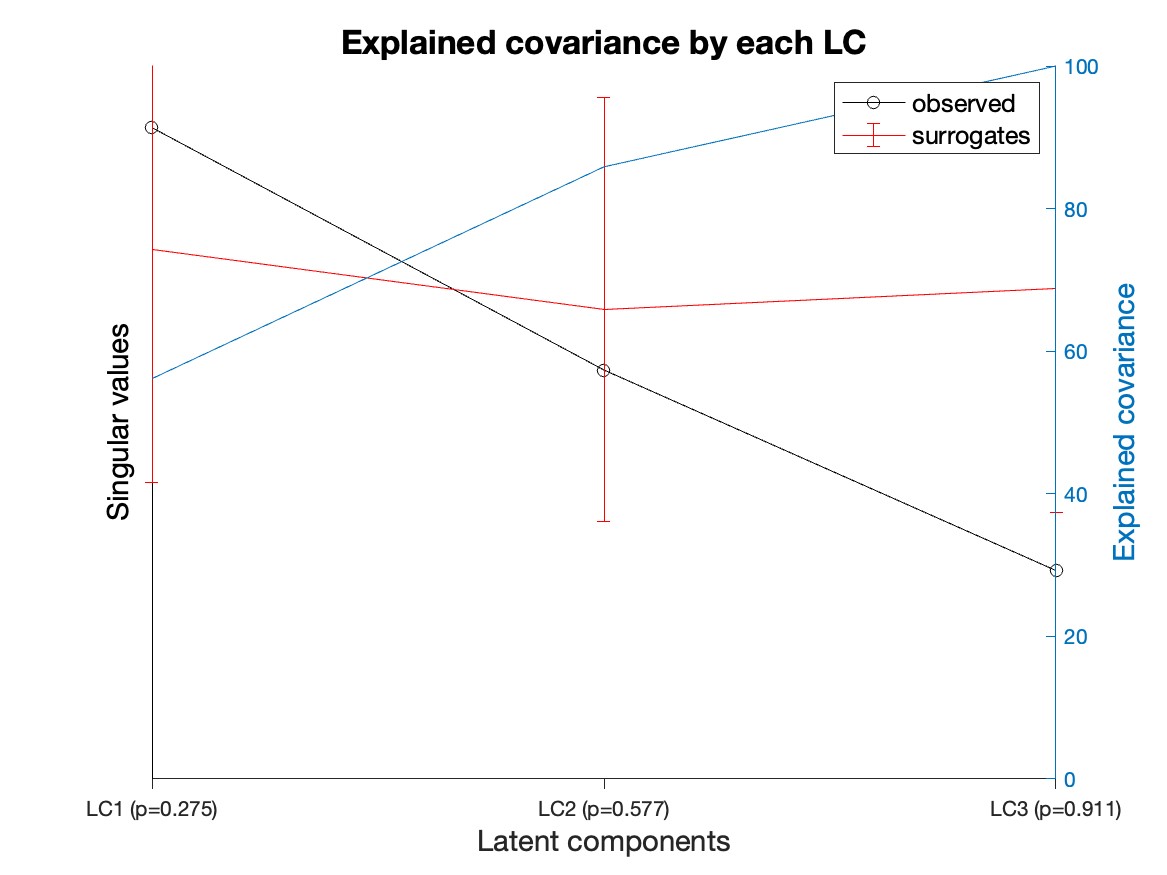
*

C


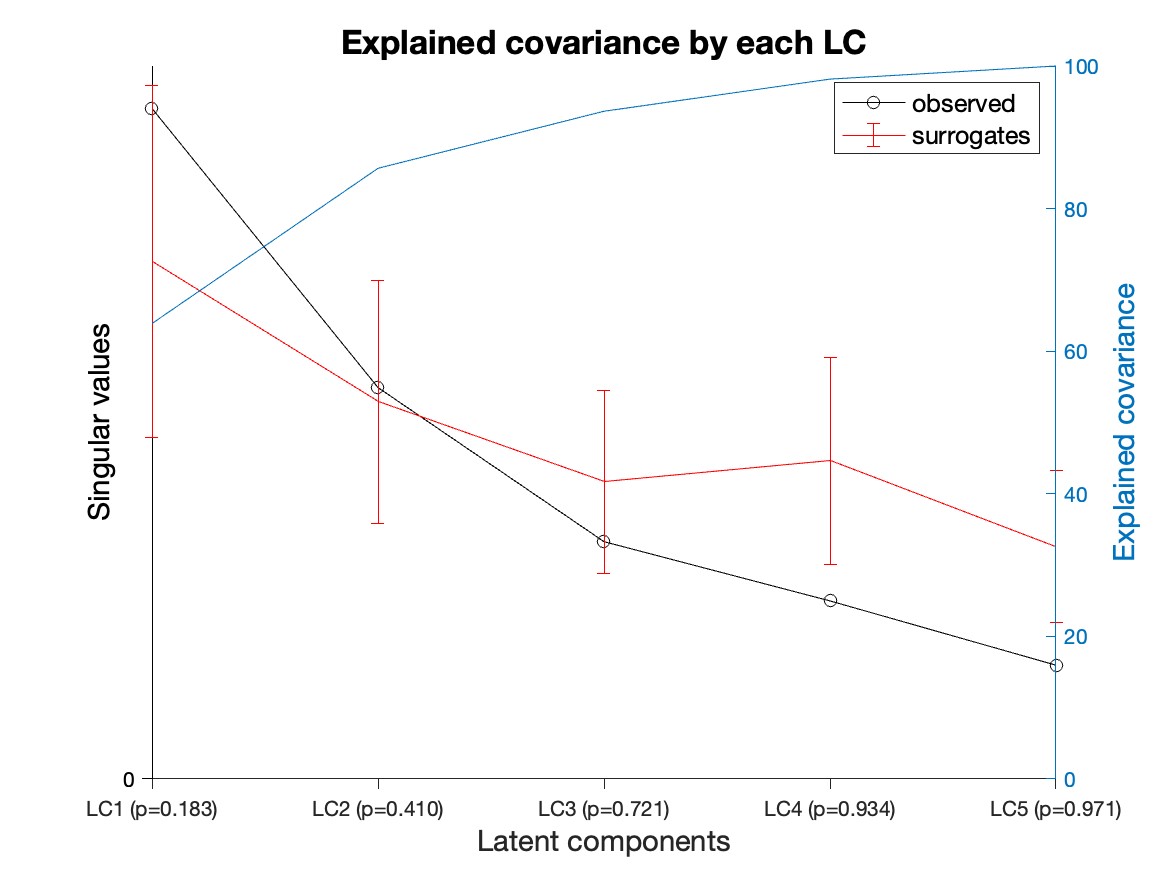


**Supplementary Fig. B.3. Explained covariance by each latent component (LC; i.e., optimally weighted linear combinations).** Explained covariance by each LC from (A) PLSC in CPP and HC with the following biopsychosocial measures: peg-algometry (finger, ear and mean), SF-36 (physical and social functioning), perceived stress, α-amylase, cortisol, and childhood trauma; (B) PLSC in HC with the following measures: peg-algometry (finger, ear and mean), SF-36 (physical and social functioning), perceived stress, α-amylase, cortisol, and childhood trauma; (C) PLSC in CPP with the following biopsychosocial measures: WPI, Pain Severity and Interference, Subjective symptom load and symptom duration. Black circles represent the observed singular values for each LC, while red lines with error bars show the mean and variability of singular values obtained from permuted (surrogate) data under the null hypothesis. The blue line indicates the cumulative explained covariance across components. P-values (shown below each LC) reflect the statistical significance of each component based on permutation testing. No LC was statistically significant.

**B.3.2 Destrieux Atlas**

*B.3.2.1 PLSC in CPP and HC with the following biopsychosocial measures: peg-algometry (finger, ear, and mean), SF-36 (physical and social functioning), perceived stress, α-amylase, cortisol, and childhood trauma.*

*
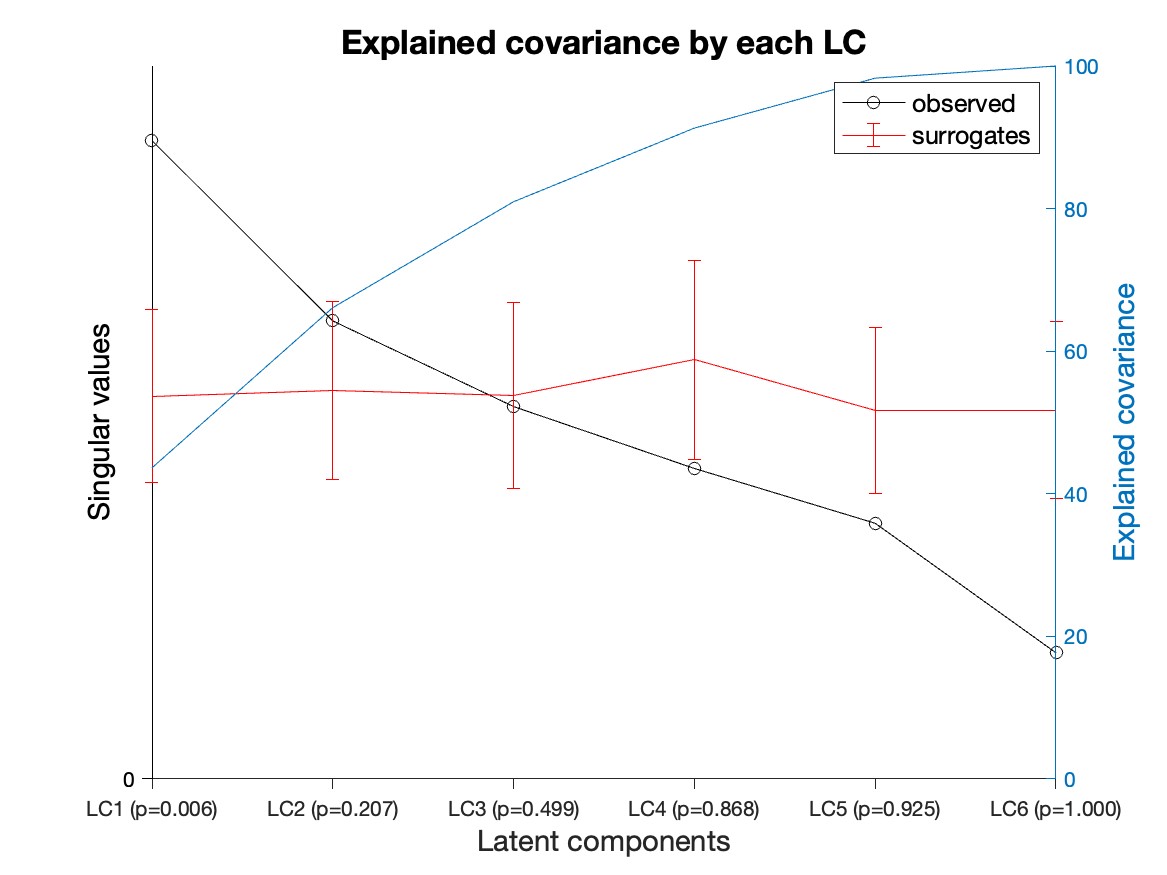
*

**Supplementary Fig. B.4. Explained covariance by each latent component (LC; i.e., optimally weighted linear combinations).** Black circles represent the observed singular values for each LC, while red lines with error bars show the mean and variability of singular values obtained from permuted (surrogate) data under the null hypothesis. The blue line indicates the cumulative explained covariance across components. P-values (shown below each LC) reflect the statistical significance of each component based on permutation testing. LC1 was statistically significant (*p* = 0.006) and accounted for the largest proportion of shared variance between the datasets.


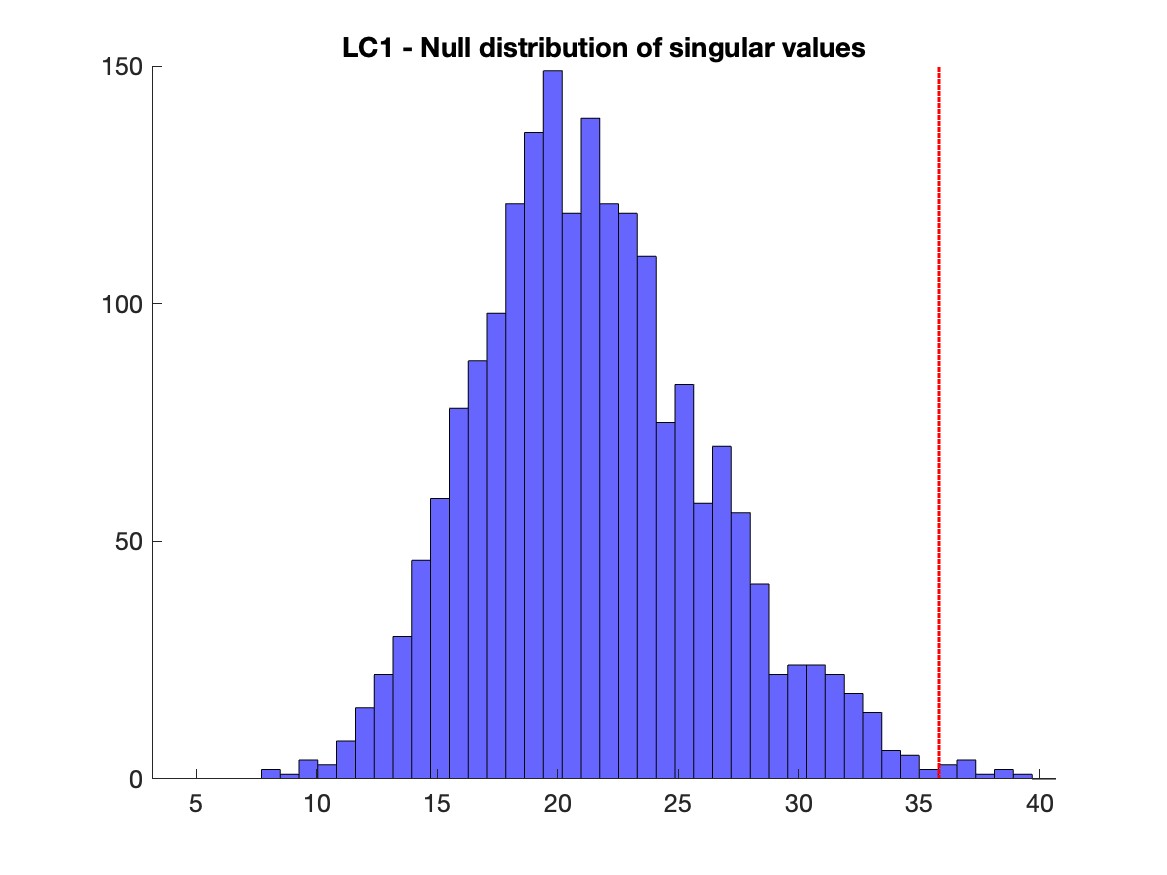


**Supplementary Fig. B.5. Permutation-based null distribution of singular values for the significant latent component.** The histogram shows the distribution of singular values obtained from 2000 permutations under the null hypothesis of no association between the datasets. The red line indicates the observed singular value from the original (unpermuted) data. The x-axis represents singular values, and the y-axis indicates their frequency across permutations. The observed value falls outside the null distribution, indicating that the LC1 is statistically significant (*p* = 0.006).

**Supplementary Table B.6. Exact values of the mean bootstrap weights, along with the lower and upper bounds of the 95% confidence intervals (CIs), are reported for the statistically significant PLSC component (LC1; *p* = 0.006). Significant rows are shown in bold.**

| **PLS Component** |  | **Mean bootstrapped weights** | **Lower CI** | **Upper CI** |
| --- | --- | --- | --- | --- |
| **LC1** | Imaging Salience | **0.568525789074653**  **-0.372001970839199**  **-0.351236305225696**  **-0.430878156877773**  **-0.401573196978527**  **-0.260977010738982** | **0.389327601832839**  **-0.503291914447811**  **-0.465958571352208**  **-0.551608148316159**  **-0.527707279803562**  **-0.373980874906274** | **0.684253320980189**  **-0.198158619100063**  **-0.127204568262845**  **-0.258079616807068**  **-0.246132403592784**  **-0.0806413354647965** |
| **LC1** | Behavioral/Design Salience | -0.0815924651384261  0.192461948297488  0.0791847994811048  0.00979141619290254  -0.0181850122066145  -0.30448763917812  0.0410780352963662  -0.0841883963323332  -0.0035600859562915  0.121365649940193  -0.0358354561614636  0.0268081690126539  0.16445429210361  0.207066630333478  **-0.348782725363011**  **-0.70185074897695**  0.0958742405918004  **-0.371502039910285** | -0.387684382414211  -0.0026220880767901  -0.139561567602698  -0.272348851327515  -0.326892846632157  -0.463419288666642  -0.203648754587989  -0.254564524248736  -0.207632952503876  -0.145855298396808  -0.243227297261651  -0.151934170229929  -0.221977953734672  -0.168339231864651  **-0.50375746801617**  **-0.741045877397655**  -0.21939342626848  **-0.497351923441086** | 0.154286068901805  0.332836818154306  0.253561334430524  0.315227502546633  0.240323928921987  0.0414523613452401  0.240631851122056  0.116931479544028  0.306261017227692  0.301251917425086  0.221978149732127  0.232064370080368  0.350195802254796  0.43268261705391  **-0.0610352388788406**  **-0.381722405867604**  0.382501717295646  **-0.122241318807899** |

*B.3.2.2 PLSC in HC with the following measures: peg-algometry (finger, ear, and mean), SF-36 (physical and social functioning), perceived stress, α-amylase, cortisol, and childhood trauma.*


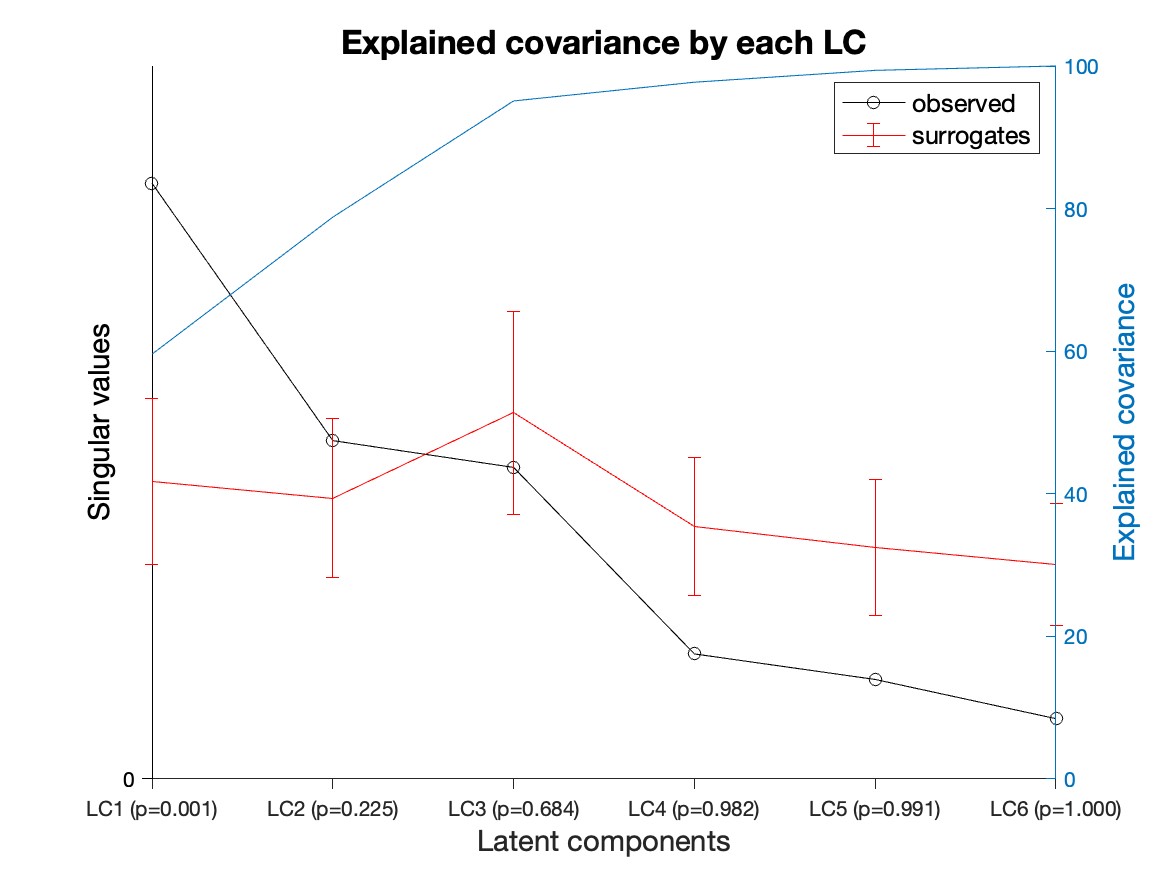


**Supplementary Fig. B.6. Explained covariance by each latent component (LC; i.e., optimally weighted linear combinations).** Black circles represent the observed singular values for each LC, while red lines with error bars show the mean and variability of singular values obtained from permuted (surrogate) data under the null hypothesis. The blue line indicates the cumulative explained covariance across components. P-values (shown below each LC) reflect the statistical significance of each component based on permutation testing. LC1 was statistically significant (*p* = 0.001) and accounted for the largest proportion of shared variance between the datasets.

**
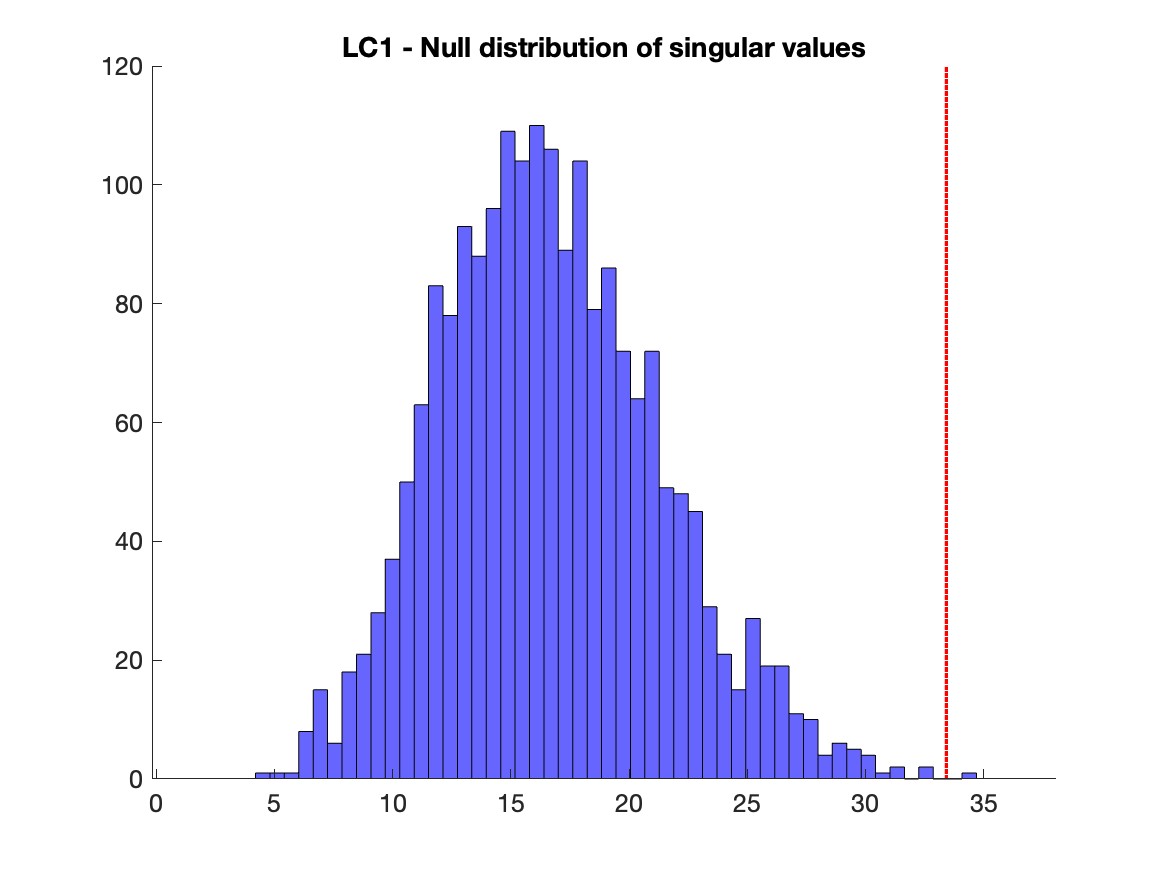
**

**Supplementary Fig. B.7. Permutation-based null distribution of singular values for the significant latent component.** The histogram shows the distribution of singular values obtained from 2000 permutations under the null hypothesis of no association between the datasets. The red line indicates the observed singular value from the original (unpermuted) data. The x-axis represents singular values, and the y-axis indicates their frequency across permutations. The observed value falls outside the null distribution, indicating that the LC1 is statistically significant (*p* = 0.001).

**Supplementary Table B.7. Exact values of the mean bootstrap weights, along with the lower and upper bounds of the 95% confidence intervals (CIs), are reported for the statistically significant PLSC component (LC1; p = 0.001). Significant rows are shown in bold.**

| **PLS Component** |  | **Mean bootstrapped weights** | **Lower CI** | **Upper CI** |
| --- | --- | --- | --- | --- |
| **LC1** | Imaging Salience | **0.558768342716069**  **-0.36515469780401**  **-0.373087707114397**  **-0.342506238831269**  **-0.368380290331265**  **-0.40277907928197** | **0.340362653699092**  **-0.516865811040447**  **-0.499074228038479**  **-0.481396910724391**  **-0.509854914118452**  **-0.533340091663108** | **0.704264893238453**  **-0.198345046117299**  **-0.0911146516529413**  **-0.149047710793793**  **-0.219299191724814**  **-0.162966389342698** |
| **LC1** | Behavioral/Design Salience | 0.140788722441737  -0.0187313672821064  0.0472059673486852  0.213186579438665  0.209694879660618  **-0.360770065892943**  **-0.753785482639494**  0.0949257198688151  **-0.42522972776599** | -0.125671191517092  -0.256744336985564  -0.103649361453297  -0.116407930558607  -0.114020330994525  **-0.474318823885666**  **-0.805524866749779**  -0.185691427535486  **-0.590249770170933** | 0.363359390434818  0.217652312743418  0.199327162220705  0.390778042386393  0.433428266704559  **-0.162979113010009**  **-0.499183604779529**  0.306261019817664  **-0.202388511965881** |

*
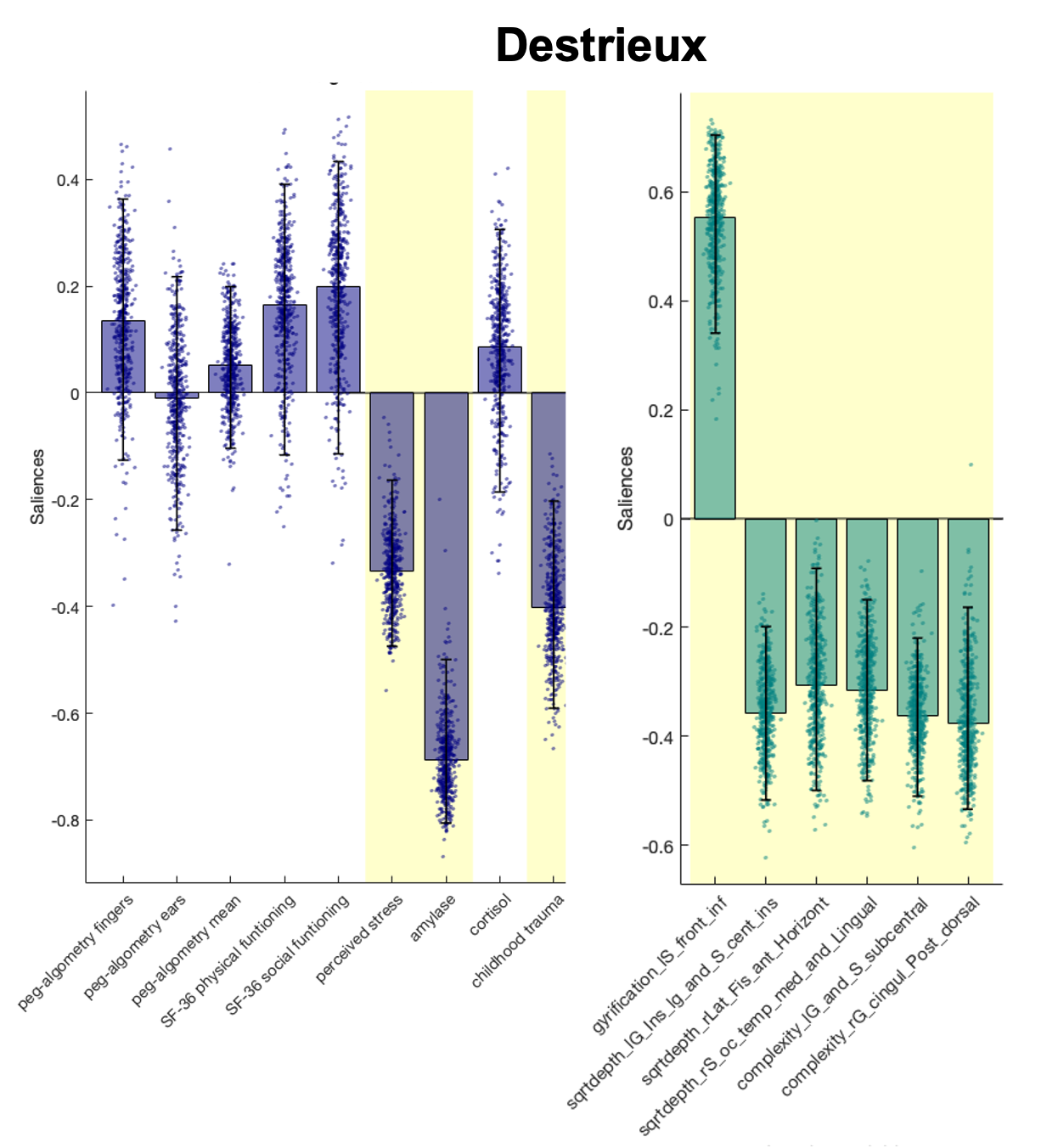
*

**Supplementary Figure B.8: Partial Least Squares (PLS) correlation analysis of CPP-related biopsychosocial characteristics and surface-based morphometry (SBM) measures in HC only.** The latent component (LC; optimally weighted linear combinations) for CPP-related biopsychosocial characteristics and SBM measures derived from the Destrieux atlas. Salience weights from the significant components (*p*_LC-Destrieux_ = 0.001) indicate each variable’s contribution to the multivariate pattern. Bars represent salience weights. Error bars indicate the 5^th^–95^th^ percentile range of the bootstrap distribution. Yellow shading marks salience weights that were both statistically significant and robust (i.e., stable across bootstrap resamples; indicated by dots). As all variables were standardised, salience weights can be interpreted similarly to correlation coefficients: variables with weights in the same direction contribute to a positive association, whereas weights in opposite directions indicate an inverse association. Childhood trauma was assessed with the Childhood Trauma Questionnaire (CTQ) and perceived stress with the Perceived Stress Scale (PSS). α-amylase refers to salivary α-amylase concentration (U/mL). Cortisol refers to the cortisol awakening response (CAR), expressed as AUC_I_. Stress biomarker measures were adjusted for age, sex, hormonal contraception, menstrual cycle phase, and smoking status. Peg algometry scores were adjusted for age, sex, psychotropic medication, and non-opioid analgesics. Brain-region saliences correspond to the significant regions shown in Figure 2, and were adjusted for age, sex, and a mood composite score.

*B.3.2.3 PLSC in CPP with the following biopsychosocial measures: WPI, Pain Severity and Interference, Subjective symptom load, and symptom duration.*

*
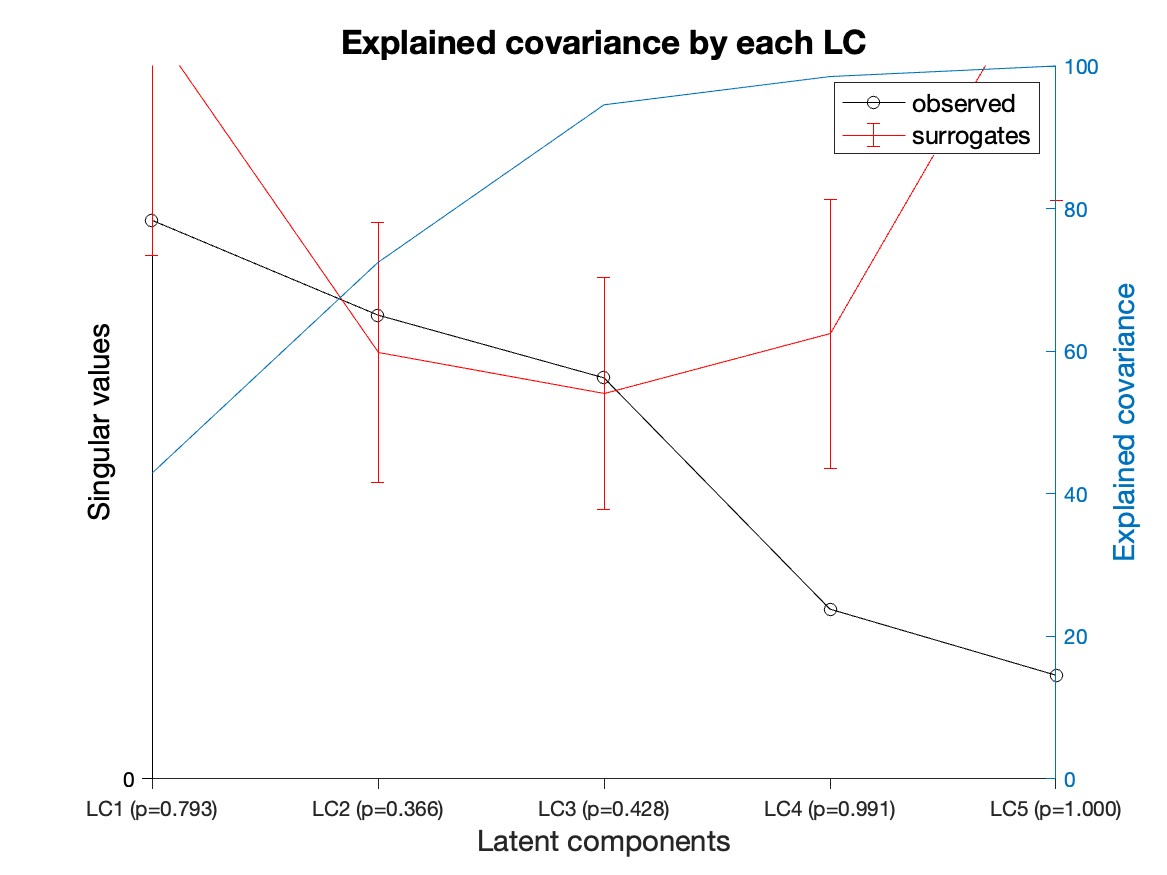
*

**Supplementary Fig. B.9. Explained covariance by each latent component (LC; i.e., optimally weighted linear combinations).** Black circles represent the observed singular values for each LC, while red lines with error bars show the mean and variability of singular values obtained from permuted (surrogate) data under the null hypothesis. The blue line indicates the cumulative explained covariance across components. P-values (shown below each LC) reflect the statistical significance of each component based on permutation testing. No LC was statistically significant.

**B.3.3 Schaefer Atlas**

*B.3.3.1 PLSC in CPP and HC with the following biopsychosocial measures: peg-algometry (finger, ear and mean), SF-36 (physical and social functioning), perceived stress, α-amylase, cortisol, and childhood trauma.*

*
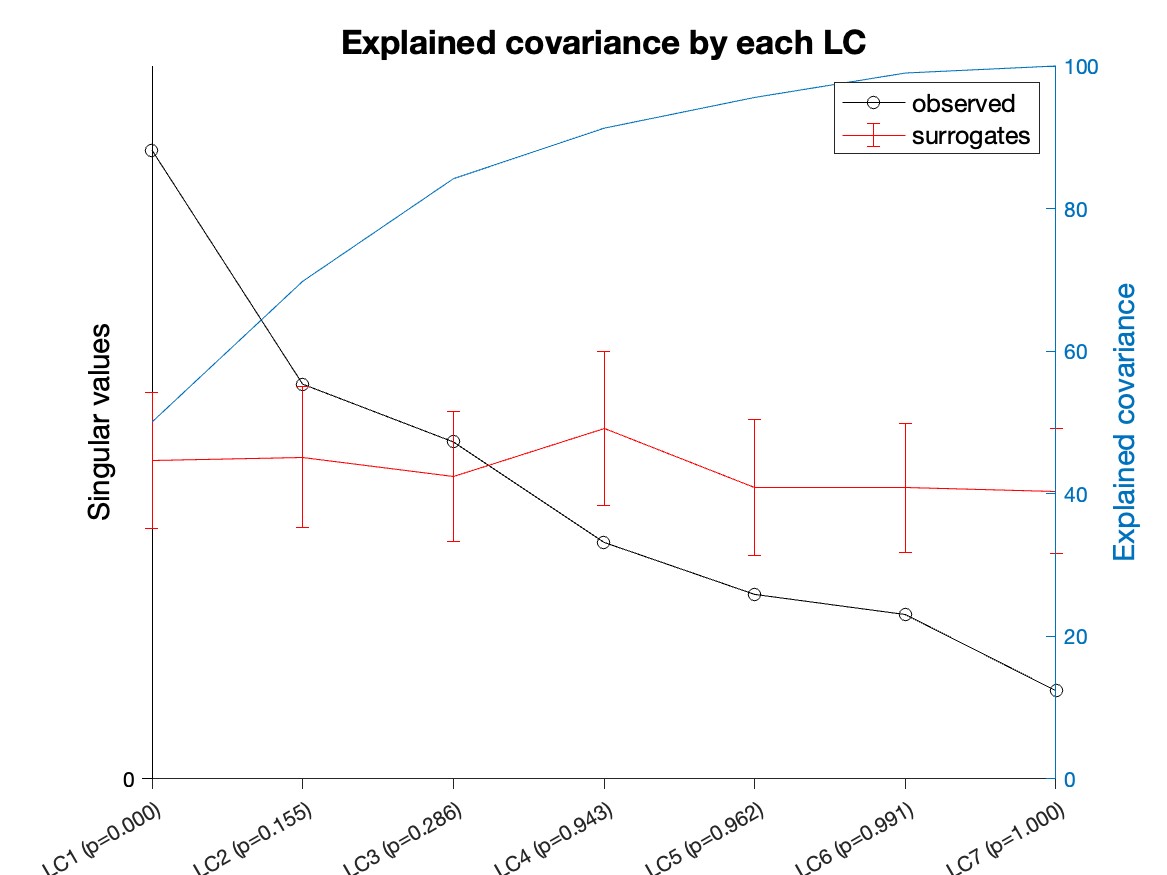
*

**Supplementary Fig. B.10. Explained covariance by each latent component (LC; i.e., optimally weighted linear combinations).** Black circles represent the observed singular values for each LC, while red lines with error bars show the mean and variability of singular values obtained from permuted (surrogate) data under the null hypothesis. The blue line indicates the cumulative explained covariance across components. P-values (shown below each LC) reflect the statistical significance of each component based on permutation testing. LC1 was statistically significant (*p* < 0.001) and accounted for the largest proportion of shared variance between the datasets.


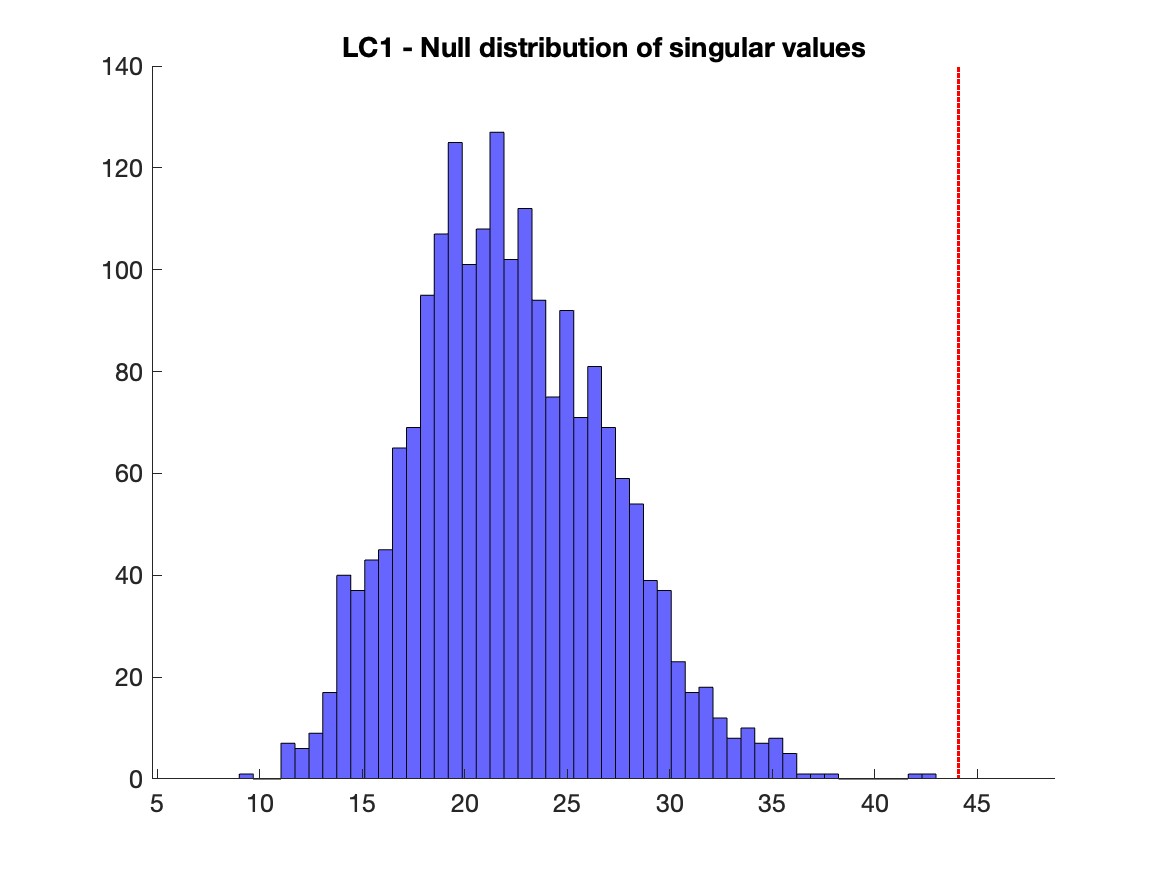


**Supplementary Fig. B.11. Permutation-based null distribution of singular values for the significant latent component.** The histogram shows the distribution of singular values obtained from 2000 permutations under the null hypothesis of no association between the datasets. The red line indicates the observed singular value from the original (unpermuted) data. The x-axis represents singular values, and the y-axis indicates their frequency across permutations. The observed value falls outside the null distribution, indicating that the LC1 is statistically significant (*p* < 0.001).

**Supplementary Table B.8. Exact values of the mean bootstrap weights, along with the lower and upper bounds of the 95% confidence intervals (CIs), are reported for the statistically significant PLSC component (LC1; *p* < 0.001). Significant rows are shown in bold**.

| **PLS Component** |  | **Mean bootstrapped weights** | **Lower CI** | **Upper CI** |
| --- | --- | --- | --- | --- |
| **LC1** | Imaging Salience | **0.241675919780112**  **0.582109579598705**  **0.420012980774501**  **0.388863103535535**  **-0.465824716455569**  0.0232915260226093  **-0.239959596978564** | **0.0832071791766545**  **0.378184218825581**  **0.243305072815769**  **0.242489512602089**  **-0.606576633654565**  -0.0774316792944072  **-0.366204375093505** | **0.354669013995323**  **0.680249548840494**  **0.515943314049873**  **0.471571275544664**  **-0.278003895850651**  0.184921623561834  **-0.0739859446370383** |
| **LC1** | Behavioral/Design Salience | 0.0960071385718232  0.208118264234826  0.178141778184806  0.18371651587333  0.226396424496652  **-0.426349693890009**  -0.069883393817127  **-0.40751702418213**  0.0469802532605314  -0.018520320329679  -0.0419915571587802  -0.037562505875236  0.0372917021293441  0.237396958559026  **-0.404856244361415**  **-0.489149637914453**  0.0905499820370559  -0.0561456817687563 | -0.168934772939184  -0.0083231577700449  -0.0375119904299657  -0.0833773105733661  0.0103325321576133  **-0.570853800209296**  -0.302215972333695  **-0.545906579086858**  -0.145523124854836  -0.197813646482473  -0.201721344185875  -0.1622500420728  -0.196452964166369  -0.0494565563153472  **-0.502914993503933**  **-0.590197135112736**  -0.145089772041546  -0.337379721715676 | 0.302613575818719  0.355617067271899  0.323536534384974  0.33245557391894  0.390266633681995  **-0.062891333602544**  0.172161926142613  **-0.211014650443012**  0.24190667065841  0.152586462935534  0.150667572424814  0.103765143972024  0.187947927743288  0.356390225785682  **-0.147310543856651**  **-0.249899557995515**  0.259069036815523  0.191641857349116 |

*B.3.3.2 PLSC in HC with the following measures: peg-algometry (finger, ear and mean), SF-36 (physical and social functioning), perceived stress, α-amylase, cortisol, and childhood trauma.*

*
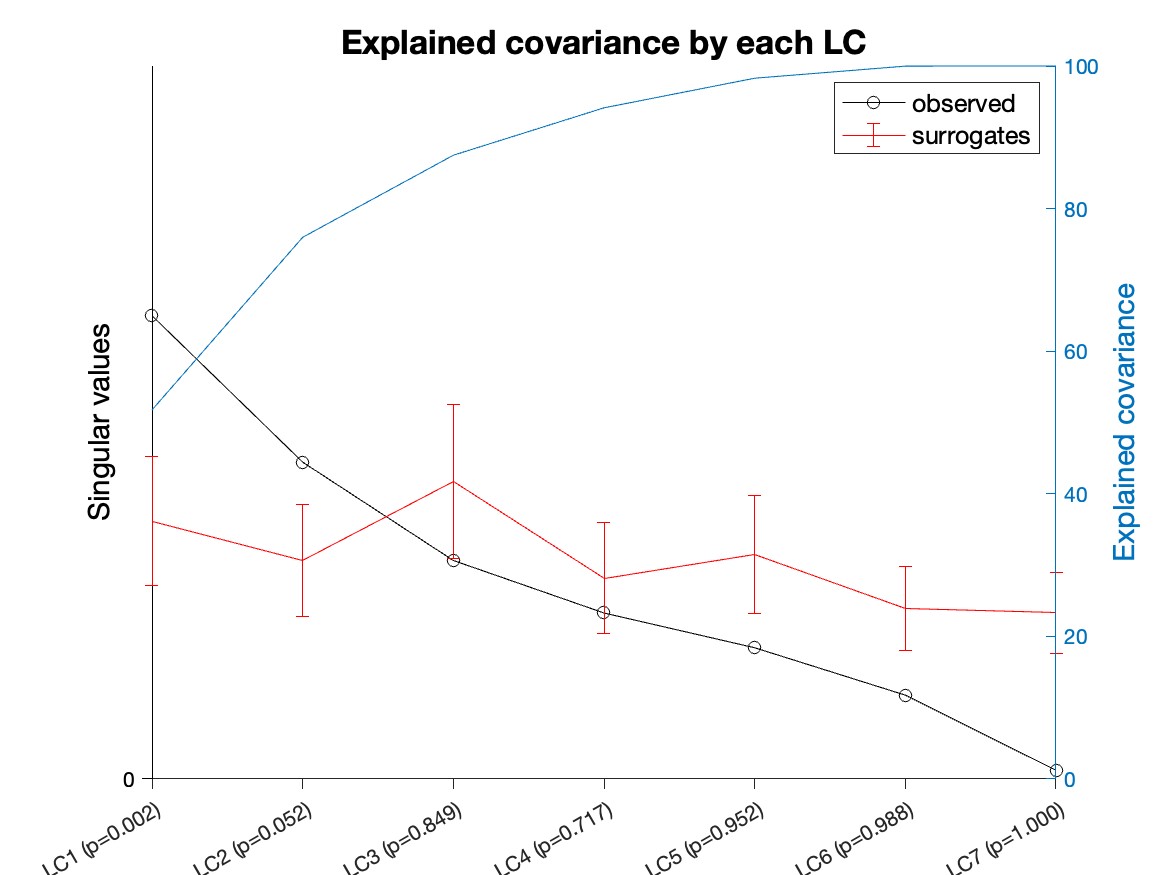
*

**Supplementary Fig. B.12. Explained covariance by each latent component (LC; i.e., optimally weighted linear combinations).** Black circles represent the observed singular values for each LC, while red lines with error bars show the mean and variability of singular values obtained from permuted (surrogate) data under the null hypothesis. The blue line indicates the cumulative explained covariance across components. P-values (shown below each LC) reflect the statistical significance of each component based on permutation testing. LC1 was statistically significant (*p* = 0.002) and accounted for the largest proportion of shared variance between the datasets.

**
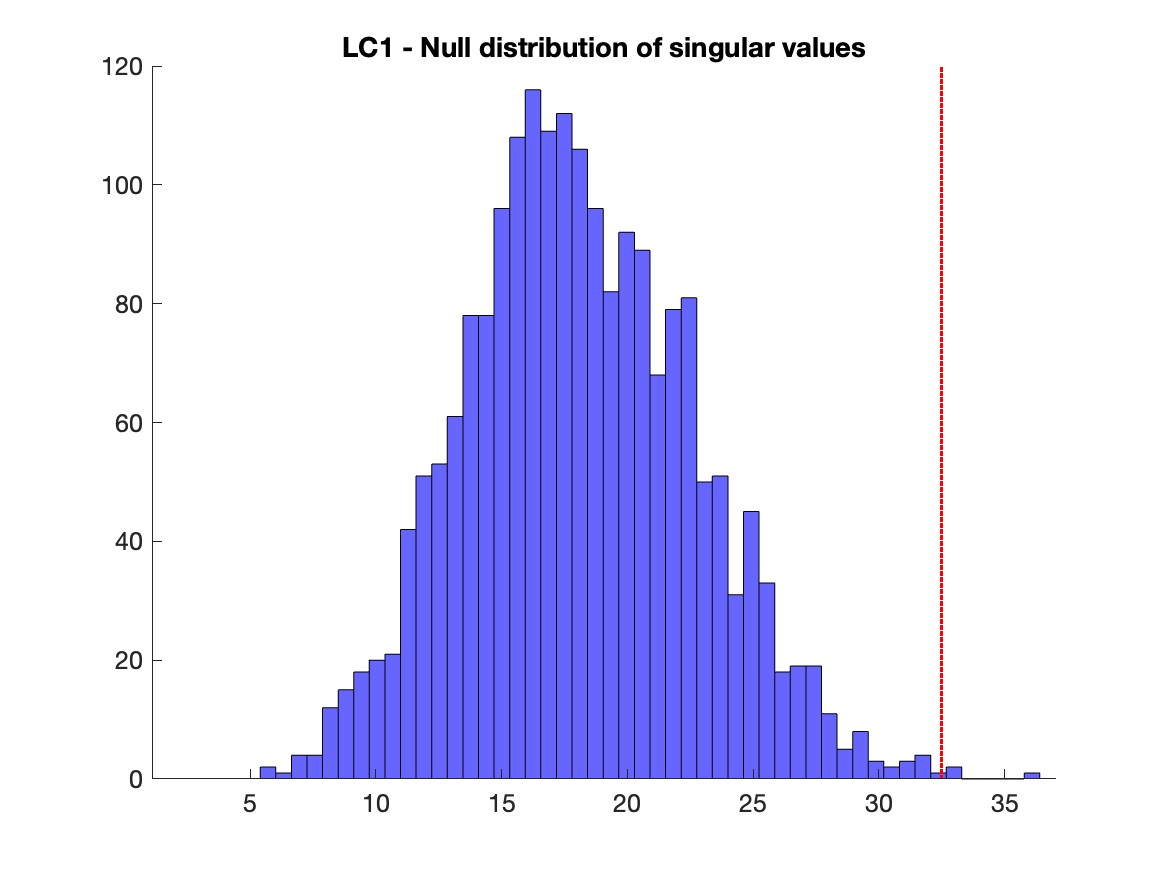
**

**Supplementary Fig. B.13. Permutation-based null distribution of singular values for the significant latent component.** The histogram shows the distribution of singular values obtained from 2000 permutations under the null hypothesis of no association between the datasets. The red line indicates the observed singular value from the original (unpermuted) data. The x-axis represents singular values, and the y-axis indicates their frequency across permutations. The observed value falls outside the null distribution, indicating that the LC1 is statistically significant (*p* = 0.002).

**Supplementary Table B.9. Exact values of the mean bootstrap weights, along with the lower and upper bounds of the 95% confidence intervals (CIs), are reported for the statistically significant PLSC component (LC1; p = 0.002). Significant rows are shown in bold.**

| **PLS Component** |  | **Mean bootstrapped weights** | **Lower CI** | **Upper CI** |
| --- | --- | --- | --- | --- |
| **LC1** | Imaging Salience | **0.177381557397822**  **0.585133123048628**  **0.338278943573213**  **0.257261978126977**  **-0.451007025773927**  **0.352031927041682**  **-0.343809289283384** | **0.00117218328034663**  **0.349422147884065**  **0.124954851102127**  **0.0690240386122133**  **-0.586976932084281**  **0.166178163796692**  **-0.522950855573662** | **0.316140304534839**  **0.687094115165387**  **0.490015821371281**  **0.361786970520591**  **-0.236245970033429**  **0.488608958810659**  **-0.122283512163018** |
| **LC1** | Behavioral/Design Salience | 0.014602111364093  -0.0775622670185485  -0.0484352860027335  -0.0532437245267292  **0.37503291554801**  **-0.601155671101378**  **-0.691215220345528**  0.0922229898288521  -0.0163894055697602 | -0.139487558977694  -0.19326647847226  -0.135745720387817  -0.240251078634193  **0.0413130070785746**  **-0.690450316708137**  **-0.770202538722764**  -0.104673598110892  -0.326161531548498 | 0.207096376263332  0.0887630647615384  0.085128155867457  0.0991117793007887  **0.556355189453219**  **-0.390984780099717**  **-0.497761988021744**  0.255632898612062  0.245062568835898 |

*
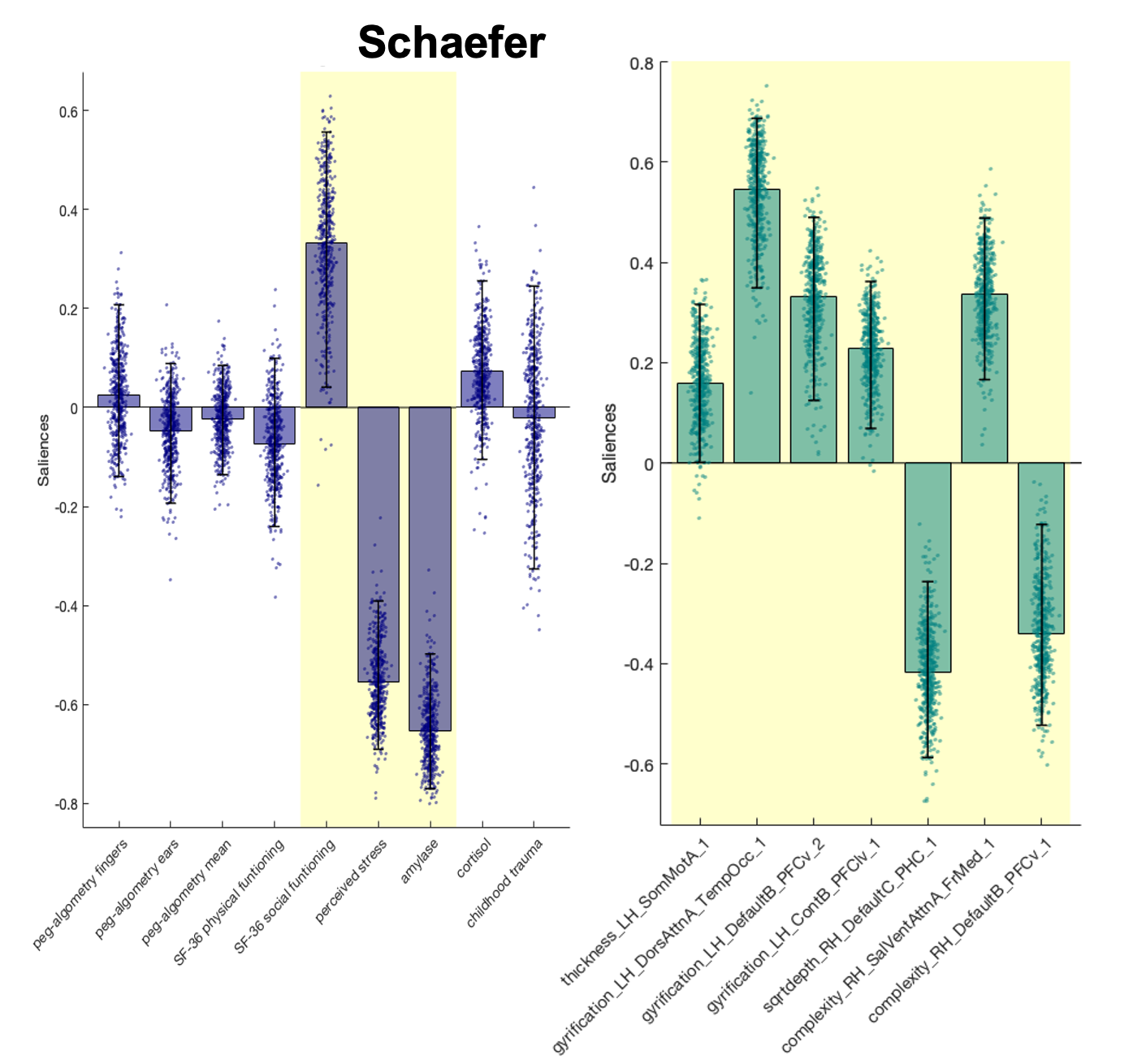
*

**Supplementary Figure B.14: Partial Least Squares (PLS) correlation analysis of CPP-related biopsychosocial characteristics and surface-based morphometry (SBM) measures in HC only.** The latent component (LC; optimally weighted linear combinations) for CPP-related biopsychosocial characteristics and SBM measures derived from the Schaefer atlas. Salience weights from the significant components (*p*_LC-Schaefer_ = 0.002) indicate each variable’s contribution to the multivariate pattern. Bars represent salience weights. Error bars indicate the 5^th^–95^th^ percentile range of the bootstrap distribution. Yellow shading marks salience weights that were both statistically significant and robust (i.e., stable across bootstrap resamples; indicated by dots). As all variables were standardised, salience weights can be interpreted similarly to correlation coefficients: variables with weights in the same direction contribute to a positive association, whereas weights in opposite directions indicate an inverse association. Childhood trauma was assessed with the Childhood Trauma Questionnaire (CTQ) and perceived stress with the Perceived Stress Scale (PSS). α-amylase refers to salivary α-amylase concentration (U/mL). Cortisol refers to the cortisol awakening response (CAR), expressed as AUC_I_. Stress biomarker measures were adjusted for age, sex, hormonal contraception, menstrual cycle phase, and smoking status. Peg algometry scores were adjusted for age, sex, psychotropic medication, and non-opioid analgesics. Brain-region saliences correspond to the significant regions shown in Figure 2, and were adjusted for age, sex, and a mood composite score.

*B.3.3.3 PLSC in CPP with the following biopsychosocial measures: WPI, Pain Severity and Interference, Subjective symptom load, and symptom duration.*

**
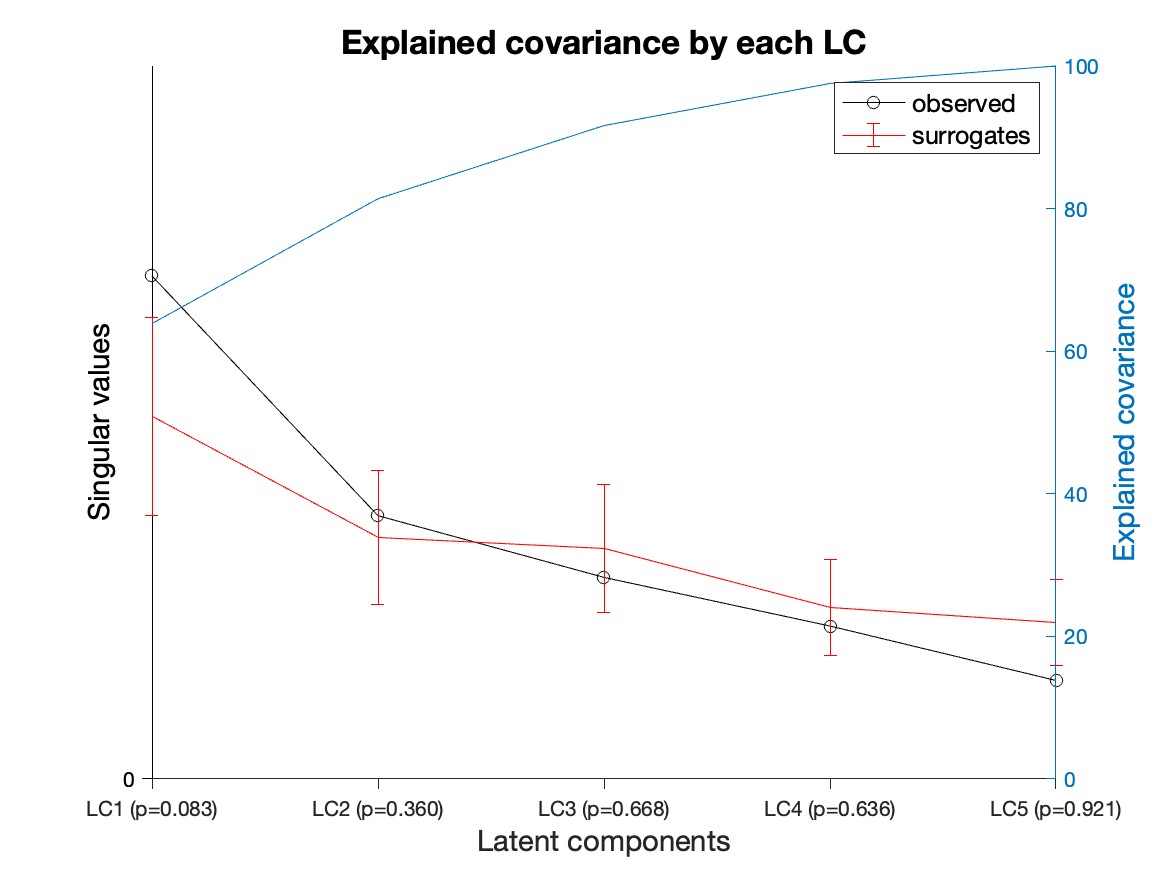
**

**Supplementary Fig. B.15. Explained covariance by each latent component (LC; i.e., optimally weighted linear combinations).** Black circles represent the observed singular values for each LC, while red lines with error bars show the mean and variability of singular values obtained from permuted (surrogate) data under the null hypothesis. The blue line indicates the cumulative explained covariance across components. P-values (shown below each LC) reflect the statistical significance of each component based on permutation testing. No LC was statistically significant.

**B.4 Sensitivity analyses of group differences in ROI-wise group differences in Surface-Based Morphometry measures under alternative covariate adjustment.**

Supplementary Table B.10.: Sensitivity analyses of ROI-wise group differences in morphometric measures using the DK40, Destrieux, and Schaefer atlases.

|  | **Model covariates** | **Atlas** | **Contrast** | **Region** | **Hemisphere** | **T** | ***p* value** |
| --- | --- | --- | --- | --- | --- | --- | --- |
| **Gyrification** | Minimal (age, sex) | DK40 | CPP>HC | Pars orbitalis  Pars opercularis  Pars triangularis  Fusiform | left  left  left  right | 2.921  2.873  2.444  3.357 | 0.025  0.023  0.018  0.011 |
|  |  | Destrieux | CPP>HC  CPP<HC | G_and_S_cingul-Ant  G_temp_sup-G_T_transv | left  left | 2.961  2.313 | 0.043  0.024 |
|  |  | Schaefer | CPP>HC | 17Networks_LH_DefaultB_PFCv_2 | left | 4.077 | 0.006 |
|  | Extended (age, sex, mood composite, psychotropic medication) | DK40 | - | - | - | - | - |
|  |  | Destrieux | CPP > HC | S_front_inf | left | 3.034 | 0.015 |
|  |  | Schaefer | CPP > HC  CPP > HC | 17Networks_LH_DorsAttnA_TempOcc_1  17Networks_LH_DefaultB_PFCv_2 | left  left | 3.449  2.297 | 0.018  0.042 |
| **Sulcal depth** | Minimal (age, sex) | DK40 | - | - | - | - | - |
|  |  | Destrieux | - | - | - | - | - |
|  |  | Schaefer | - | - | - | - | - |
|  | Extended (age, sex, mood composite, psychotropic medication) | DK40 | CPP < HC | lateralorbitofrontal | right | 3.315 | 0.012 |
|  |  | Destrieux | CPP < HC  CPP < HC  CPP < HC | S_circular_insula_ant  S_circular_insula_inf  G_temp_sup-Plan_polar | right  right  right | 3.399  2.720  2.547 | 0.007  0.018  0.021 |
|  |  | Schaefer | CPP < HC | 17Networks_RH_LimbicA_TempPole_1 | right | 2.110 | 0.020 |
| **Fractal dimension** | Minimal (age, sex) | DK40 | CPP < HC | lateraloccipital | right | 2.864 | 0.029 |
|  |  | - | - | - | - | - | - |
|  |  | - | - | - | - | - | - |
|  | Extended (age, sex, mood composite, psychotropic medication) | DK40 | CPP > HC | temporalpole | right | 2.549 | 0.048 |
|  |  | Destrieux | CPP < HC | G_and_S_subcentral | left | 4.012 | 0.002 |
|  |  | Schaefer | CPP < HC  CPP < HC | 17Networks_LH_SomMotB_S2_2  17Networks_RH_SomMotB_S2_2 | left  left | 3.601  2.790 | 0.035  0.044 |
| **Thickness** | Minimal (age, sex) | DK40 | CPP<HC | lateralorbitofrontal | left | 2.926 | 0.010 |
|  |  | Destrieux | CPP<HC | G_orbital | left | 2.662 | 0.020 |
|  |  | Schaefer | - | - | - | - | - |
|  | Extended (age, sex, mood composite, psychotropic medication) | DK40 | - | - | - | - | - |
|  |  | Destrieux | - | - | - | - | - |
|  |  | Schaefer | CPP > HC | 17Networks_LH_SomMotA_1 | left | 3.742 | 0.015 |

Results were adjusted for multiple comparisons using the Holm–Bonferroni method.

**B.5 Pain Distribution in CPP**

**Supplementary Table B.10: Pain sites in patients with CPP.**

| **Pain Sites** | Cases (%) out of 30 patients with CPP |
| --- | --- |
| Head  Neck and shoulder area  Right arm  Left arm  Chest  Abdomen  Back  Pelvis  Right leg  Left leg | 20 (66.7%)  27 (90%)  16 (53.3%)  16 (53.3%)  8 (26.7%)  17 (56.7%)  24 (80%)  10 (33.3%)  22 (73.3%)  18 (60%) |

**C. References**

[1.](https://www.zotero.org/google-docs/?WXWmcr) [Spielberger C, Gorsuch R, Lushene R, Vagg P, Jacobs G. *Manual for the State-Trait Anxiety Inventory*. IV. CA:Consulting Psychologists Press; 1983.](https://www.zotero.org/google-docs/?WXWmcr)

[2.](https://www.zotero.org/google-docs/?WXWmcr) [Beck AT, Ward CH, Mendelson M, Mock J, Erbaugh J. An Inventory for Measuring Depression. *Archives of General Psychiatry*. 1961;4(6):561-571. doi:10.1001/archpsyc.1961.01710120031004](https://www.zotero.org/google-docs/?WXWmcr)

[3.](https://www.zotero.org/google-docs/?WXWmcr) [Shahid A, Wilkinson K, Marcu S, Shapiro CM, eds. Leeds Sleep Evaluation Questionnaire (LSEQ). In: *STOP, THAT and One Hundred Other Sleep Scales*. Springer New York; 2012:211-213. doi:10.1007/978-1-4419-9893-4](https://www.zotero.org/google-docs/?WXWmcr)

[4.](https://www.zotero.org/google-docs/?WXWmcr) [Wolfe F, Clauw DJ, Fitzcharles MA, et al. The American College of Rheumatology Preliminary Diagnostic Criteria for Fibromyalgia and Measurement of Symptom Severity. *Arthritis Care & Research*. 2010;62(5):600-610. doi:10.1002/acr.20140](https://www.zotero.org/google-docs/?WXWmcr)

[5.](https://www.zotero.org/google-docs/?WXWmcr) [Radbruch L, Loick G, Kiencke P, et al. Validation of the German Version of the Brief Pain Inventory. *Journal of Pain and Symptom Management*. 1999;18(3):180-187. doi:10.1016/S0885-3924(99)00064-0](https://www.zotero.org/google-docs/?WXWmcr)

[6.](https://www.zotero.org/google-docs/?WXWmcr) [Ware JE, Sherbourne CD. The MOS 36-item short-form health survey (SF-36). I. Conceptual framework and item selection. *Med Care*. 1992;30(6):473-483.](https://www.zotero.org/google-docs/?WXWmcr)

[7.](https://www.zotero.org/google-docs/?WXWmcr) [Klinitzke G, Romppel M, Häuser W, Brähler E, Glaesmer H. Die deutsche Version des Childhood Trauma Questionnaire (CTQ) – psychometrische Eigenschaften in einer bevölkerungsrepräsentativen Stichprobe. *PPmP - Psychotherapie · Psychosomatik · Medizinische Psychologie*. 2011;62:47-51. doi:10.1055/s-0031-1295495](https://www.zotero.org/google-docs/?WXWmcr)

[8.](https://www.zotero.org/google-docs/?WXWmcr) [Cohen S, Kamarck T, Mermelstein R. A Global Measure of Perceived Stress. *Journal of Health and Social Behavior*. 1983;24(4):385-396. doi:10.2307/2136404](https://www.zotero.org/google-docs/?WXWmcr)

[9.](https://www.zotero.org/google-docs/?WXWmcr) [Häuselmann S, Wyss A, Weber S, et al. Anterior insular co-activation patterns associated with stress markers in chronic primary pain. *Brain Communications*. Published online April 2026. doi:10.1093/braincomms/fcag121](https://www.zotero.org/google-docs/?WXWmcr)

[10.](https://www.zotero.org/google-docs/?WXWmcr) [Golden SH, Wand GS, Malhotra S, Kamel I, Horton K. Reliability of hypothalamic–pituitary–adrenal axis assessment methods for use in population-based studies. *Eur J Epidemiol*. 2011;26(7):511-525. doi:10.1007/s10654-011-9585-2](https://www.zotero.org/google-docs/?WXWmcr)

[11.](https://www.zotero.org/google-docs/?WXWmcr) [Wust S, Wolf J, Hellhammer DH, Federenko I, Schommer N, Kirschbaum C. The cortisol awakening response - normal values and confounds. *Noise and Health*. 2000;2(7):79-88.](https://www.zotero.org/google-docs/?WXWmcr)

[12.](https://www.zotero.org/google-docs/?WXWmcr) [Stalder T, Kirschbaum C, Kudielka BM, et al. Assessment of the cortisol awakening response: Expert consensus guidelines. *Psychoneuroendocrinology*. 2016;63:414-432. doi:10.1016/j.psyneuen.2015.10.010](https://www.zotero.org/google-docs/?WXWmcr)

[13.](https://www.zotero.org/google-docs/?WXWmcr) [Ali N, Nater UM. Salivary Alpha-Amylase as a Biomarker of Stress in Behavioral Medicine. *IntJ Behav Med*. 2020;27(3):337-342. doi:10.1007/s12529-019-09843-x](https://www.zotero.org/google-docs/?WXWmcr)

[14.](https://www.zotero.org/google-docs/?WXWmcr) [Pruessner JC, Kirschbaum C, Meinlschmid G, Hellhammer DH. Two formulas for computation of the area under the curve represent measures of total hormone concentration versus time-dependent change. *Psychoneuroendocrinology*. 2003;28(7):916-931. doi:10.1016/s0306-4530(02)00108-7](https://www.zotero.org/google-docs/?WXWmcr)

[15.](https://www.zotero.org/google-docs/?WXWmcr) [Egloff N, Klingler N, von Känel R, et al. Algometry with a clothes peg compared to an electronic pressure algometer: a randomized cross-sectional study in pain patients. *BMC Musculoskelet Disord*. 2011;12:174. doi:10.1186/1471-2474-12-174](https://www.zotero.org/google-docs/?WXWmcr)

[16.](https://www.zotero.org/google-docs/?WXWmcr) [Desikan RS, Ségonne F, Fischl B, et al. An automated labeling system for subdividing the human cerebral cortex on MRI scans into gyral based regions of interest. *NeuroImage*. 2006;31(3):968-980. doi:10.1016/j.neuroimage.2006.01.021](https://www.zotero.org/google-docs/?WXWmcr)

[17.](https://www.zotero.org/google-docs/?WXWmcr) [Gninenko N, Müller E, Aybek S. Reduced microstructural white matter integrity is associated with the severity of physical symptoms in functional neurological disorder. *NeuroImage: Clinical*. 2025;46:103791. doi:10.1016/j.nicl.2025.103791](https://www.zotero.org/google-docs/?WXWmcr)

[18.](https://www.zotero.org/google-docs/?WXWmcr) [Glasser MF, Sotiropoulos SN, Wilson JA, et al. The minimal preprocessing pipelines for the Human Connectome Project. *NeuroImage*. 2013;80:105-124. doi:10.1016/j.neuroimage.2013.04.127](https://www.zotero.org/google-docs/?WXWmcr)

[19.](https://www.zotero.org/google-docs/?WXWmcr) [Sotiropoulos SN, Jbabdi S, Xu J, et al. Advances in diffusion MRI acquisition and processing in the Human Connectome Project. *NeuroImage*. 2013;80:125-143. doi:10.1016/j.neuroimage.2013.05.057](https://www.zotero.org/google-docs/?WXWmcr)

[20.](https://www.zotero.org/google-docs/?WXWmcr) [Smith RE, Tournier JD, Calamante F, Connelly A. SIFT: Spherical-deconvolution informed filtering of tractograms. *NeuroImage*. 2013;67:298-312. doi:10.1016/j.neuroimage.2012.11.049](https://www.zotero.org/google-docs/?WXWmcr)

[21.](https://www.zotero.org/google-docs/?WXWmcr) [Rubinov M, Sporns O. Complex network measures of brain connectivity: Uses and interpretations. *NeuroImage*. 2010;52(3):1059-1069. doi:10.1016/j.neuroimage.2009.10.003](https://www.zotero.org/google-docs/?WXWmcr)

[22.](https://www.zotero.org/google-docs/?WXWmcr) [Hedges LV. Distribution Theory for Glass’s Estimator of Effect Size and Related Estimators. *Journal of Educational Statistics*. 1981;6(2):107-128. doi:10.2307/1164588](https://www.zotero.org/google-docs/?WXWmcr)
